# Advanced Ultrasound Energy Transfer Technologies using Metamaterial Structures

**DOI:** 10.1002/advs.202401494

**Published:** 2024-06-18

**Authors:** Iman M. Imani, Hyun Soo Kim, Joonchul Shin, Dong‐Gyu Lee, Jiwon Park, Anish Vaidya, Chowon Kim, Jeong Min Baik, Yu Shrike Zhang, Heemin Kang, Sunghoon Hur, Hyun‐Cheol Song

**Affiliations:** ^1^ Electronic Materials Research Center Korea Institute of Science and Technology (KIST) Seoul 02792 Republic of Korea; ^2^ Department of Materials Science and Engineering Korea University Seoul 02841 Republic of Korea; ^3^ School of Advanced Materials Science and Engineering Sungkyunkwan University (SKKU) Suwon 16419 Republic of Korea; ^4^ KIST‐SKKU Carbon‐Neutral Research Center Sungkyunkwan University (SKKU) Suwon 16419 Republic of Korea; ^5^ Division of Engineering in Medicine Department of Medicine Brigham and Women's Hospital Harvard Medical School Cambridge MA 02139 USA; ^6^ KHU‐KIST Department of Converging Science and Technology Kyung Hee University Seoul 02447 Republic of Korea

**Keywords:** metamaterials, nanogenerators, piezoelectric, triboelectric, ultrasound, wireless energy transfers

## Abstract

Wireless energy transfer (WET) based on ultrasound‐driven generators with enormous beneficial functions, is technologically in progress by the valuation of ultrasonic metamaterials (UMMs) in science and engineering domains. Indeed, novel metamaterial structures can develop the efficiency of mechanical and physical features of ultrasound energy receivers (US‐ETs), including ultrasound‐driven piezoelectric and triboelectric nanogenerators (US‐PENGs and US‐TENGs) for advantageous applications. This review article first summarizes the fundamentals, classification, and design engineering of UMMs after introducing ultrasound energy for WET technology. In addition to addressing using UMMs, the topical progress of innovative UMMs in US‐ETs is conceptually presented. Moreover, the advanced approaches of metamaterials are reported in the categorized applications of US‐PENGs and US‐TENGs. Finally, some current perspectives and encounters of UMMs in US‐ETs are offered. With this objective in mind, this review explores the potential revolution of reliable integrated energy transfer systems through the transformation of metamaterials into ultrasound‐driven active mediums for generators.

## Graphical abstract

With the quick growth of ultrasound energy transfer technology based on triboelectric and piezoelectric nanogenerators (TENGs and PENGs), the recent progress in achieving efficient energy harvesting is presented, with a review of design strategies through understanding the influence and significance of ultrasonic metamaterials concept in ultrasound‐driven TENG and PENG devices for current applications.

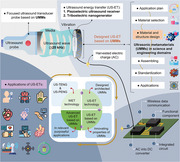

## 1 Introduction

The rapid growth of innovative nanogenerator technologies has accelerated the research into ultrasound‐based energy harvesting applications; an effectively distinctive improvement of nanogenerators is the investigation of ultrasonic metamaterials (UMMs).^[^ ^1^, ^2^ ^]^ Metamaterials have emerged as a fascinating area of promising applications in various fields of electromagnetic, mechanical, thermal, and acoustic while this paper principally focuses on reviewing the pivotal role of UMMs in ultrasound energy transfers (US‐ETs) as a focal technology within this domain. UMMs are engineered materials capable of manipulating ultrasound waves based on their design, rather than solely relying on their constituent materials, which are typically not found in nature. UMMs hold significant importance for energy harvesting by creating structural designs to convert ultrasound energy into electrical energy efficiently. Functional structures of UMMs are inspiring for enhancing the performance of mechanical and physical properties of power generators such as flexibility, stretchability, sensitivity, selectivity, stability, and vibratory.^[^ ^2^, ^3^, ^4^, ^5^, ^6^ ^]^ One approach to designing UMMs for energy harvesting involves seamlessly integrating piezoelectric or triboelectric materials and ultrasonic resonant properties.^[^ ^7^ ^]^ This approach helps develop piezoelectric nanogenerators (PENGs) and triboelectric nanogenerators (TENGs) that can convert the mechanical energy of ultrasound waves into electrical energy. Notably, the amplitude increase and frequency optimization of ultrasound waves can enhance the resonant property and improve the efficiency of US‐ETs.^[^ ^8^ ^]^ For example, using UMM‐based probe‐lens can focus ultrasound waves onto the active region of the generator and improve power output efficiency by amplifying mechanical vibrations.^[^ ^9^ ^]^ Furthermore, a variation of practical metamaterials and innovative design has promoted the performance of generators, including increased feasibility, mechanical adaptability, and response/recovery period.^[^ ^9^, ^10^ ^]^ The applications of US‐ETs are transpired in various features of power harvesting such as self‐power implantable and wearable devices, underwater wireless communication, and robotics. In this article, we have reviewed the recent advancements of UMMs in ultrasound‐driven PENGs and TENGs (US‐PENGs and US‐TENGs) for energy harvesting to practical applications. Initially, the fundamentals, classification, and design engineering of UMMs are introduced after talking about the significance of ultrasound energy in WET technology. Next, the recent significant progress of UMMs in US‐PENGs and US‐TENGs is covered. Additionally, the innovative strategies of UMMs on the application of US‐PENGs and US‐TENGs are briefly summarized. In the end, some trend standpoints and challenges for the possible future development of UMMs in US‐ETs are also discussed.

**Figure 1:**
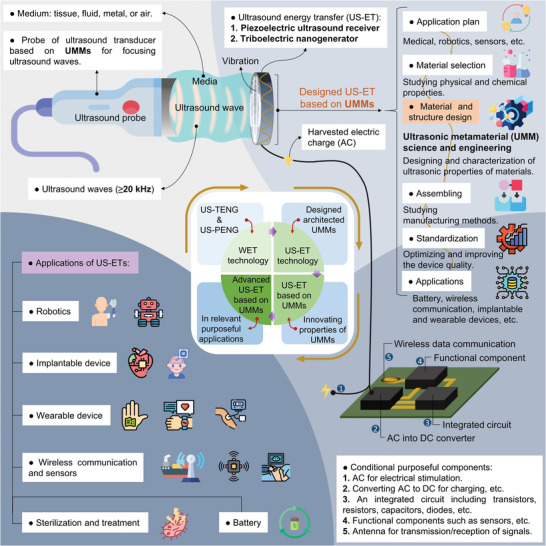
A prime linkage view of US‐ET technology with UMMs science and engineering.

For more details, **Figure** **1** presents a comprehensive vibe from the contents of this review article which is classified into four principal sections. First, it refers to delivering ultrasound waves remotely through an ultrasound transducer probe toward receivers that could be piezoelectric or triboelectric generators. The vibration energy will be changed to electrical energy/signal via US‐ETs for functionalizing. Here, we considered the implementation steps of a US‐ET which included planning application and strategy, material selection, structure design of materials, assembling, standardization and optimization, and application. In the materials and structure design section, the knowledge of UMM is showing a significant role in engineering and manufacturing a US‐ET with a smart structure and design, and we are focusing on this concept more. The harvested energy/signal can be functionalized by an electronic circuit with the components for the purposeful applications listed. In light of recent developments of WETs, it is evident that US‐ETs stand as beneficial technology. This review aims to explore the latest advancements and applications in the realm of US‐ET technology with particular emphasis on their synergy with innovation driving of metamaterials.

## 2 Ultrasound for Wireless Energy Transfer Technology

**Figure 2:**
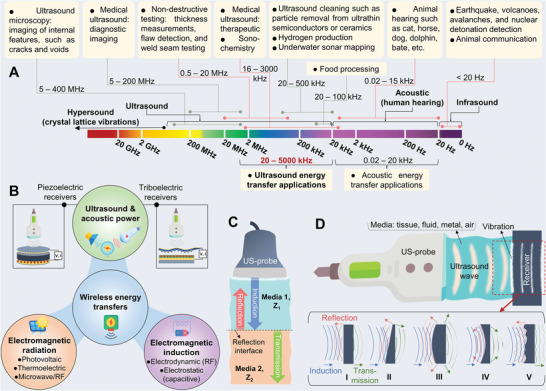
A) The approximated frequency ranges of the sound energy spectrum with different sources and applications. B) The technology of wireless energy transfers (WETs), the WETs based on ultrasound are divided into triboelectric and piezoelectric receivers. C) Illustration schematic for the interaction of vertical ultrasound waves with the interface of receiver‐media including induction, reflection, and transmission in the interface of two media with different impedances. D) Illustration schematic for the ultrasound wave shapes after collision with the interface of receiver‐media including (I) flat surface, (II) angled surface, (III) convex surface, (IV) concave surface, (V) rough surface.

Sound waves are forms of longitudinal mechanical waves generated by the vibration of a source such as musical instruments; they propagate through a medium including gases, liquids, and solids; however, they differ in frequency ranges and applications. The frequency ranges of sound with diverse applications in daily life and industry are presented in **Figure** **2**A. This includes the infrasound range (<20 Hz), the acoustic or human hearing range (20 Hz–20 kHz), the ultrasound range (20 kHz–1 GHz), and the hypersound range (>GHz).^[^ ^11^, ^12^, ^13^ ^]^ The ability to manipulate and utilize sound waves has led to advancements in various life experiences and communications;^[^ ^14^ ^]^ for example, sound navigation and ranging (sonar) systems use ultrasound waves to determine and map the depth and location of objects underwater.^[^ ^15^, ^16^, ^17^ ^]^

Ultrasound waves refer to sound waves with frequencies higher than the upper boundary of human hearing, which is practically considered to be around the typical range from 20 kHz to 100 megahertz (MHz), reportedly in previous papers.^[^ ^14^ ^]^ Ultrasound energy transducers generate an ultrasound with a wide range of frequency and power for numerous applications. The core element of an ultrasound transducer is one or more piezoelectric crystals with unique properties for converting electrical energy into mechanical vibrations for applications in various fields. Ultrasound is a valuable tool in medicine, industry, and scientific research due to its non‐invasive nature, real‐time imaging capabilities, and ability to penetrate various substances.^[^ ^18^, ^19^, ^20^, ^21^ ^]^ For example, in medicine, ultrasound imaging is known as sonography, which uses high‐frequency sound waves to create images of the body's internal structures.^[^ ^22^ ^]^ It is commonly applied for diagnostic purposes, such as examining organs, monitoring fetal development during pregnancy, and echocardiography, to visualize the heart's structure and function.^[^ ^23^ ^]^ Additionally, high‐intensity focused ultrasound is used for therapeutic purposes, such as tumor ablation, targeted drug delivery, lithotripsy (breaking kidney stones), and physical therapy.^[^ ^24^ ^]^ Beyond medical use, ultrasound plays a crucial role in non‐destructive testing techniques. It allows the inspection of the integrity and defects of materials, fluid flow rates, and cracks without causing damage.^[^ ^25^, ^26^ ^]^ Moreover, ultrasound helps determine the physical properties of materials, such as density, elasticity, and thickness.^[^ ^27^, ^28^ ^]^ It is also employed in cleaning processes by creating microscopic bubbles to remove contaminants from objects.^[^ ^29^, ^30^ ^]^ Furthermore, ultrasound contributes to optimizing vehicle acoustics to reduce noise and vibration inside car cabins and enhancing features like parking assistance systems, collision avoidance systems, and blind‐spot detection.^[^ ^31^, ^32^ ^]^ Last, ultrasound aids in exploring subsurface structures in geophysics, such as oil and gas reservoirs or geological formations.^[^ ^33^, ^34^ ^]^

The technology of WETs can be classified based on different energy sources including electromagnetic radiation (thermal and optical), electromagnetic induction (capacitive coupling and inductive coupling), and sound (acoustic and ultrasound),^[^ ^35^, ^36^ ^]^ as shown in Figure 2B. The WET technology remotely transmits different energy forms from the power sources onto or into a receiver to convert into electric energy without physical wired connections for a wide range of applications. Comparing US‐ET technology with other technologies can provide valuable insights into its advantages, limitations, and unique capabilities. WETs based on ultrasound energy possess advantages including non‐invasive remote mechanical energy, available power source from transducers, and feasibility in short and long distances; these benefits make them suitable for environments with safety concerns such as medical applications. Compared to other WET technologies, for example, even though photovoltaic energy transfers include a renewable energy source, it may not be consistently available in all indoor conditions and feasible due to limited permeability. In addition, thermoelectric or pyroelectric devices directly convert significant temperature differentials into electrical energy for large‐scale applications without moving parts, however, they typically exhibit low efficiency due to the limitation of their widespread adoption and penetration for power generation compared to US‐ET technology. Also, radio frequency power transfer technologies which are electromagnetic induction coupling technic, can achieve high efficiency in short distances by exposing high levels of radiation but that may raise health concerns in the medical fields. Furthermore, capacitive coupling is an electrostatic energy transfer system with sufficient efficiency in power transfer in short distances power transmission by inducing high voltages of the electric field that may pose safety damages.^[^ ^37^ ^]^

Ultrasonic wave, in the typical range of frequency of a few kHz to several MHz, has been explored as a potential remote energy source for WET, specifically for short‐range and low‐power energy harvesting applications including wireless charging of small electronic devices, medical implants, sensors, and Internet of Things (IoT) devices.^[^ ^37^, ^38^, ^39^, ^40^, ^41^ ^]^ The first application of US‐ET was presented in 1985 by Cochran et al. through a piezoelectric implant for osteogenesis.^[^ ^42^ ^]^ The concept involves converting electrical energy into ultrasound waves by a transmitter, which is then received and converted back into electrical energy by a US‐ET device.^[^ ^38^, ^39^ ^]^ However, notably, the US‐ET is still an emergent field and practical implementations are limited.

Calculating the intensity or power calculation of ultrasound is crucial for optimizing ultrasound levels to achieve high accuracy or technological advancements. This is particularly critical in the medical field to ensure efficiency and compliance with safety regulations to prevent potential harm to patients. Accurate calculations help avoid adverse biological effects and potential damage to human organs, caused by high‐intensity ultrasound.^[^ ^20^, ^43^, ^44^ ^]^ The pressure of ultrasound can be defined by Pascal (Pa) which is a force of one newton per square meter. In addition, a metric logarithmic‐scale of sound‐pressure level (SPL) is usually used as an effective measurement index by the equation of $\mathrm{SPL}\;=\;20\;\log\frac{P}{P_{rf}}$ and unit is decibel (dB), where *P_rf_* is the pressure of the lowest sound we can hear in the air that is 2  ×  10^−5^ Pa and in the water is 1  ×  10^−6^ Pa. The ultrasound intensity (*I*) of continuous wave in the media is determined through Equation (1):

(1) $$I=\frac{P^{2}}{2\rho c}$$

where *P* is the amplitude of the ultrasound pressure, *ρ* is the density of the media, and *c* is the sound speed in the media. The unit of ultrasound power is typically defined as watts per square meter (W m^−2^).^[^ ^45^ ^]^ Through employing certain power/voltage to a commercial transducer, the ultrasound power efficiency can be computed by Equations (2) and (3):

(2) $$\begin{array}{c} \mathrm{Efficiency}\;=100\times \frac{P_{in}}{P_{out}}\;\left(in\;percent\right)=\;10\;\log\frac{P_{in}}{P_{out}}\left(in\;decibels\right) \end{array}$$

*P_in_* and *P_out_* are the average power consumption of the transducer and generated power from the receiver, respectively. Based on Joule's Law, in AC circuits where the voltage and current are not in phase, additional factors such as angular frequency need to be considered to calculate the true power. Via applying expansion of Fourier sin series for square waves, the power consumption (*P*_*in* − *square*_) can be calculated by below equation:

(3) $$\begin{array}{cc} P_{in-square} & =\frac{1}{2}\;{\left(\frac{4}{\pi }\right)}^{2}\sum_{n\;=\;1,3,5,\text{…}}^{\infty }{\left(\frac{V_{\circ }}{n}\right)}^{2}G\;\left(n\omega_{\circ }\right)\\ & =\frac{1}{2}\;{\left(\frac{4}{\pi }\right)}^{2}[{\left(V_{\circ }\right)}^{2}G\left(\omega_{\circ }\right)+{\left(\frac{V_{\circ }}{3}\right)}^{2}G\left(3\omega_{\circ }\right)\\ & \;+{\left(\frac{V_{\circ }}{5}\right)}^{2}G\left(5\omega_{\circ }\right)+\cdots ] \end{array}$$

$V_{\circ }$ is the voltage amplitude, $\omega_{\circ }$ is angular frequency, and *G* is conductance.^[^ ^46^ ^]^

The efficiency of US‐ET depends on some factors such as the distance between the transmitter and receiver, the frequency of ultrasound waves, and the material design of the transducers and receivers.^[^ ^47^ ^]^

The frequency of ultrasound waves used in US‐ET can vary depending on the specific application. Higher frequencies generally allow for smaller transducer sizes and better spatial resolution but may have shorter propagation distances. Lower frequencies can achieve longer transmission distances but require larger transducers.^[^ ^48^, ^49^ ^]^ To improve the power transfer efficiency and overcome the inherent divergence of ultrasound waves, focusing techniques can be employed. These techniques use specialized transducer designs or acoustic lenses to concentrate the ultrasound energy into a narrower beam, allowing for better power transfer over longer distances.^[^ ^24^, ^48^ ^]^

Ultrasound propagation media can be classified into three groups: plastic/tissue/fluid, metals, and air. Among these, metal media groups have garnered the majority of research attention in industrial applications, while publications on ultrasound through the air are fairly limited due to low density and low sound impedance.^[^ ^47^, ^50^, ^51^, ^52^, ^53^, ^54^, ^55^ ^]^ A substantial portion of US‐ET studies deal with biomedical applications, particularly in powering implantable devices or facilitating direct energy conversion (without intermediate conversion) to electrical energy. Since the characteristic impedance of tissue is comparable to that of water, experiments are often conducted in a fluid medium rather than using actual tissue.^[^ ^47^, ^56^, ^57^, ^58^ ^]^ Ultrasound waves can penetrate media or receiver with the sinusoidal cycle of rarefaction (low density) and compression (high density) phases and interact physically with media or receiver composites.^[^ ^14^, ^59^ ^]^ Therefore, understanding the nature of the materials, alongside the structural design of the receiver, is essential for precise wave confinement or interference, requiring accurate alignment between the transmitter and receiver to achieve high‐efficiency US‐ET.^[^ ^60^ ^]^

The crucial role of the receiver's material and design tuning using metamaterials can be highlighted by elucidating the interactions of vertical ultrasound waves with the media boundaries of the receiver at different surface structure modes. When vertical ultrasound waves (at 90°) interact with the interface of receiver‐media, several phenomena can occur, including reflection, transmission, and absorption. A part of the ultrasound can be reflected toward the source medium or in a changed direction the amount of reflection and transmission depends on the impedance mismatch between the two media or receiver media.^[^ ^61^ ^]^

Ultrasound impedance (*Z*) is a physical property that represents the resistance of a medium to the passage of sound waves and is given by the density (*ρ*) and sound velocity (*c*) of media, (*Z = ρ × c*) and the unit of *Z* is defined MPa s m^−1^ = MRayl. The sound velocity (*c = λ × f*) remains relatively constant for a specific medium in a stable temperature range but we must consider that increasing frequency (*f*) of ultrasound waves generally encounter higher acoustic impedance in a medium due to their shorter wavelengths (*λ*).^[^ ^62^, ^63^ ^]^ For example, these effects have practical implications in medical ultrasound imaging that higher‐frequency ultrasound waves can provide better resolution because of their shorter wavelengths, but they also have reduced penetration capabilities due to increased attenuation and energy loss.^[^ ^14^, ^61^, ^64^, ^65^, ^66^, ^67^, ^68^, ^69^, ^70^, ^71^, ^72^, ^73^ ^]^

If there is a significant difference in sound impedance between the two media, a substantial amount of the ultrasound wave will be reflected. Furthermore, if the sound impedance is similar, more of the wave energy will be transmitted through the interface in the same direction or a different direction (refraction). The reflection coefficient (*R*) is calculated following *R = *[(*Z_2_ – Z_1_*) / (*Z_2_ + Z_1_*)]*^2^*, where *Z*_1_ is the sound impedance of the first medium (like tissue/fluid/metal/air) and *Z*_2_ is the sound impedance of the second medium or receiver (Figure 2C).^[^ ^69^, ^74^ ^]^ The reflection coefficient ranges from −1 (total reflection) to 1 (no reflection). Hereby, selecting the materials composite of US‐ET is an essential step to optimize the propagation of ultrasound waves.^[^ ^75^ ^]^ Some of the ultrasound energy can be absorbed by the receiver medium as the wave travels through it. The amount of absorption (conversion of sound energy into heat or vibration) depends on the density, viscosity, and attenuation of the media.^[^ ^66^ ^]^ The impedance mismatch of the media‐receiver interface can be reduced by adjusting the frequency and intensity of the ultrasound, using coupling agents (e.g., ultrasound gel), or optimizing the structure of the receiver by employing metamaterials.^[^ ^76^ ^]^ The structures can respond or be active conditionally under a tuned frequency and intensity of ultrasound waves. The architected US‐ET under the pressure of ultrasound waves will get reciprocating microscopic deformation due to strain–stress. The activation mechanisms of the embedded integrated materials in the US‐ET are considered for the desired response, and functional structures of UMMs hold a promise to attract ultrasound waves as much as possible and heighten mechanic–electric conversion ability.

Here, we simply explain the physical phenomena of sound interaction with structural materials plus their unique properties and potential applications. As shown in Figure 2D, the direction and shape of the ultrasound wave are changed after collision with different surface structures of a media or receiver.^[^ ^14^ ^]^ In a flat interface, the fainter wave travels back to the ultrasound beam directly and the ultrasound energy disperses on the interface equally. Whereas the reflected and transmitted waves in the other items are deformed such as rough surfaces which make scattered waves due to the surface structure mode of the receiver that shows the roles of manipulated structure/design of receivers. Based on this adventure, holographic techniques are improved through monolithic ultrasound holograms, which can reconstruct diffraction‐limited ultrasound pressure fields and thus arbitrary ultrasound beams. The complex 3D pressure and phase distributions produced by ultrasound holograms allow us to demonstrate new approaches to controlled ultrasonic manipulation of solids in water, and of liquids and solids in air for contactless transfer of power, improve medical imaging, and drive new applications of ultrasound.^[^ ^77^ ^]^

## 3 Fundamental, Classification, and Designing of Ultrasonic Metamaterials

**Figure 3:**
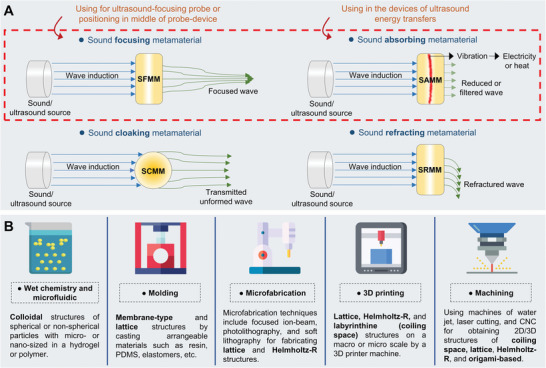
A) The classification of UMMs with their functions on sound/ultrasound wave manipulating. B) The categorization of fabrication techniques for UMMs.

Metamaterials are 3D materials constructed from artificially engineered meta‐atoms or meta‐surface structures to exhibit unique sound properties not found in natural materials.^[^ ^78^, ^79^ ^]^ These materials are designed for precise control over the propagation and manipulation of ultrasonic waves with frequencies above the upper limit of human hearing, typically above 20 kHz.^[^ ^80^ ^]^ Additionally, metamaterials have other potential applications across various fields due to their unique and engineered properties. For instance, optical and photonic metamaterials enable precise manipulation of light, facilitating innovations in photonics, imaging, and optical communication systems. Similarly, electromagnetic metamaterials find utility in antennas and wireless communications, where they can be customized to demonstrate specific electromagnetic properties.^[^ ^81^ ^]^ The surface of the UMMs exhibits unique band structures that govern the behavior of ultrasonic waves. These band structures determine the allowed and forbidden frequency ranges for wave propagation, leading to properties such as focusing, absorbing, cloaking, negative refraction, and bandgaps.^[^ ^9^, ^82^ ^]^ The behavior of the UMMs can often be described using effective medium theory, which treats the metamaterial as a homogeneous medium with effective properties.^[^ ^83^, ^84^ ^]^ The UMMs can be classified based on sound working mechanism and here are four common classifications: sound focuser, sound absorber, sound cloak, and sound refractive;^[^ ^85^, ^86^ ^]^ as parted in **Figure** **3**A which presents conceptionally some reported function and application of metamaterials to manipulate the acoustic or ultrasound waves at high frequency regarding these four classifications. The sound‐focusing metamaterials (SFMMs) are designed to concentrate sound waves by controlling the phase and amplitude of sound waves to converge at a focal point. The SFMMs are very practical in medical imaging, ultrasonic therapy, energy harvesting, and underwater acoustics.^[^ ^79^, ^87^, ^88^ ^]^

Sound absorbing metamaterials (SAMMs) are specialized to efficiently absorb specific frequency ranges of sound waves for reducing sound reflections and preventing sound transmission through them. They employ designs such as Helmholtz resonators, membrane‐based structures, or space‐coiling‐space configurations to resonate with sound waves effectively. The internal microstructures of these materials are carefully designed to manipulate sound waves and promote efficient dissipation of sound energy.^[^ ^89^ ^]^ SFMMs are usually utilized for energy harvesting, mitigating noise pollution in industrial settings, transportation, and urban environments; or enhancing sound quality in audio systems by reducing unwanted reflections and resonances.^[^ ^90^ ^]^

Sound‐cloaking metamaterials (SCMMs) are designed to control the propagation of ultrasound around a region of interest, effectively rendering it invisible to incoming waves. Indeed, An SCMM is a layer that surrounds an object that bends incident sound waves around it and reconstructs the incident waves on the opposite side.^[^ ^91^, ^92^ ^]^ By redirecting and concealing the waves, these metamaterials such as a 2D structure of Helmholtz resonators can be used for applications such as vibration isolation and acoustic imaging.^[^ ^93^, ^94^, ^95^ ^]^

Sound refracting metamaterials (SRMMs) with a refractive index can be employed for changing the direction and transferring asymmetric or refracted sound waves which is known as asymmetric acoustic transmission and includes a negative index, near‐zero index, and high index. The SRMMs have attracted metamaterials research due to their potential applications such as noise control and medical ultrasound.^[^ ^96^, ^97^, ^98^, ^99^ ^]^

Engineering and designing the UMMs involve careful consideration of the material properties, structural design, and fabrication techniques.^[^ ^81^, ^91^, ^100^ ^]^ Choosing appropriate materials is crucial for designing the UMMs. These materials should possess the desired acoustic properties, such as high stiffness, low density, and low ultrasound attenuation.^[^ ^59^, ^79^ ^]^ Common materials include metals, polymers, composites, or ceramics, with advanced options (possessing specific properties) like piezoelectric materials offering additional functionalities.^[^ ^7^, ^85^ ^]^ Unit cells serve as the fundamental building blocks of metamaterial structures in some scenarios, interacting with neighboring periodic elements or other unit cells. The geometry, size, and arrangement of the unit cells determine the overall properties of the metamaterials typically consist of repeating patterns of subwavelength elements, such as rods, plates, membranes, or resonators.^[^ ^90^, ^101^ ^]^ Computational modeling and simulation techniques such as finite element analysis or boundary element method are commonly employed to predict and optimize the ultrasound properties of the metamaterials such as wave propagation and dispersion, with different design parameters in any limitations or challenges.^[^ ^102^, ^103^ ^]^

Furthermore, the evaluation of sound properties of fabricated UMMs is crucial and involves transmission/reflection coefficients, bandgap frequencies, ultrasound scanning maps, and impedance measurements are commonly employed for technical characterization. The current reported structures of UMMs can be nearly classified into five categories including colloidal suspensions, membrane‐type, coiling space (labyrinthine), Helmholtz resonators, and origami‐based structures.^[^ ^103^, ^104^, ^105^ ^]^ Each type of structure can be employed as an exclusive manipulator of sound waves for an application or by engineering the structure of the device it can have multiple functions. The fabrication of UMMs is attractively widespread due to the development of the quality and accuracy of their functions during the fabrication process. Several fabrication techniques can be used to manufacture UMMs, including microfluidic wet‐chemistry, molding, 3D printing, microfabrication (E‐beam lithography, focused ion‐beam, photolithography, and soft lithography), and machining (water jet machine, laser cutting machine, CNC machine, and electric discharge machine).^[^ ^9^, ^104^, ^105^, ^106^ ^]^ The choice of fabrication technique depends on the desired scale, complexity, and precision of the metamaterial structure. Figure 3B shows a category of fabrication strategies for UMMs that expose a concise explanation of each method. Colloidal UMMs are typically manufactured by microfluidic technique, which is a suspension of spherical or non‐spherical particles with micro‐ or nano‐sized in a solution of hydrogel or polymer, then a colloidal structure will be obtained after curing or drying. In the molding or casting process, the materials are considered that can gain a pre‐arranged form by a mold such as resin, PDMS, or elastomers; this technique is usually used for fabricating UMMs with membrane‐type and lattice structures. The fabrication of UMMs on micro or nanoscale is a challenge, microfabrication techniques such as E‐beam lithography, focused ion‐beam, photolithography, and soft lithography are practical for fabricating lattice and Helmholtz‐R structures UMMs. Lattice, Helmholtz‐R, and labyrinthine (coiling space) structures of UMMs on a macro or micro scale can be constructed by a 3D printer machine due to the capability of this desired technique to encompass a wide of tunable size and designs of structure. A conventional method to fabricate the UMMs with high accuracy such as water jet machine, laser cutting, CNC machine, and electric discharge machine for obtaining a variety of 2D and 3D structures of coiling space, lattice, Helmholtz‐R, and origami‐based. UMMs offer unprecedented control and manipulation of ultrasonic waves, providing opportunities for developing innovative devices and systems in fields such as acoustic communications, sensing, and energy harvesting. Ongoing research in this field aims to explore new design concepts, fabrication techniques, and applications for UMMs.

## 4 Ultrasonic Metamaterials for Energy Harvesting

**Figure 4:**
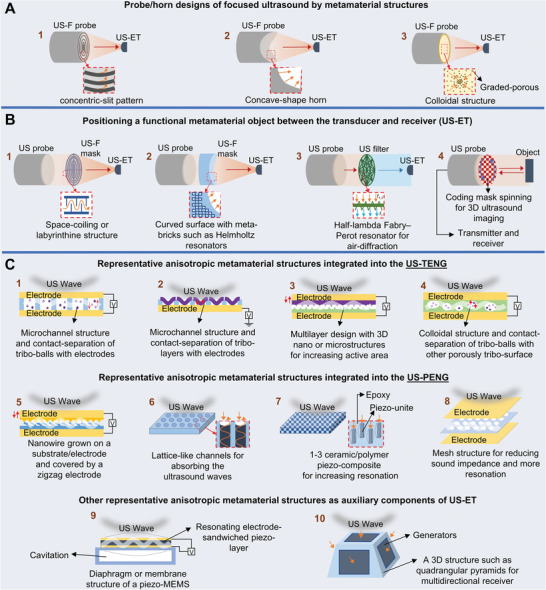
The classification of functional representative metamaterials in US‐ET systems to enhance energy harvesting capabilities including A) transducer probe/horn based on metamaterial designs, B) metamaterial positioned between the transducer and US‐ETs, C) representative anisotropic metamaterial structures integrated into the US‐ETs and other representative anisotropic metamaterial structures as auxiliary components of the US‐ETs.

UMMs offer the potential for improved energy conversion efficiency of US‐ETs by controlling ultrasound propagation/interaction and exhibiting specific resonant frequencies for selectivity enhancement in small‐scale or lightweight constraints. However, the design and fabrication of UMM structures require complex techniques and specialized materials and can exhibit limited operating bandwidth or efficiency in certain frequency ranges. Additionally, integrating UMMs into practical systems may pose integration challenges in terms of compatibility with existing infrastructure and electronic interfaces. The functional representative metamaterials with architected structures can be utilized in the US‐ET systems at different strategies that are categorized comprehensively in **Figure** **4**, including design optimization of transducer horn/probe structure, positioning a meta‐object between transducer and receiver, and metamaterial integration in the devices/systems that all of them are used to focus and scavenge energy from ambient ultrasound sources to enhance the energy harvesting capabilities of US‐ETs. The designed transducer probes based on SFMMs for the US‐ET technology domain in Figure 4A are typically attracted to concentrate sound waves toward receivers. Other strategical metamaterial structures can be positioned between the transducer and US‐ETs, which are considered for focusing, filtering, and modulating of ultrasound (Figure 4B). Additionally, Figure 4C shows the representative anisotropic structures based on SAMM integrated into the US‐ETs for enhancing the conversion of mechanical energy (sound waves) to electricity and other metamaterial structures as auxiliary components of the US‐ETs to increase the active area of the device.^[^ ^7^, ^107^ ^]^ Flexibility in metamaterials design, allowing for the creation of structures with tunable properties and enabling the adaptation of metamaterials to different operating conditions and frequencies. The research on UMMs for enhancing energy harvesting has been interesting, with ongoing efforts focused on developing novel designs by investigating different materials, geometries, and fabrication techniques. For example, a study demonstrates rational patterned electrode configurations, can maximize the net polarization difference across a piezoelectric ceramic device for elastic wave energy harvesting. To fully obtain the advantage of the amplified input elastic ultrasound wave energy by the focusing capability of a gradient‐index phononic crystal based on metamaterial structure, the voltage cancellation effect due to the coexistence of multimode strains with opposite directions was alleviated.^[^ ^88^ ^]^

SFMMs can be created as ultrasound‐focusing probes like curved piezoelectric ultrasonic transducers, space‐coiling designs, and flat annular ring arrays (by a laser ablation process to pattern the electrode of piezo) as displayed respectively in Figure 4A.^[^ ^108^ ^]^ Further structures were investigated to enhance ultrasound focusing when assembled onto ultrasound probes. A soft porous gradient‐index focusing metasurface was employed to cover a rectangular ultrasonic transducer using microfluidic techniques.^[^ ^109^ ^]^ Distance controlling of concreted sound is crucial for different application fields that can be changed by metasurface lenses. Space‐coiling sound metasurfaces have largely shown their outstanding wave manipulation capacity. Hereby, a comprehensive paradigm was presented for sound metasurfaces to extend the wave manipulations to both far fields and near fields and markedly reduce the implementation complication with a simple space‐coiling structure manufactured by the 3D printer.^[^ ^110^ ^]^

Membrane‐type sound metamaterials have undergone widespread investigation and are optimally designed to aim at ultrasound absorption. Many simple structure grating metasurface with a viscoelastic membrane were fabricated for absorption ranges of ultrasound. The main reason for absorbing the sound wave in the grating metasurface can be explained as that vibration (harmonic oscillation) from ultrasound pressure is received by the flexible absorber film (polymer layer) and the surface of the absorber can gain curvy shapes toward the inside cavity cells between metal and absorber.^[^ ^111^ ^]^ Furthermore, another research group introduced a locally resonant phononic woodpile (LRPW) that has a robust capability of underwater absorbing ultrasound in a wide frequency range. The steel woodpile layers were coated with polyurethane (PU) for more resonation and the 2D unit of LRPW can be simplified to the double‐oscillator based on the mass‐spring model.^[^ ^112^ ^]^

We offer and explain a brunch of comprehensive visions of US‐ET devices/systems for how all materials architected in Figure 4 enable tailored responsiveness.

The common feature of piezoelectric and triboelectric materials is generating AC by converting mechanical energy to electricity. However, specialized ultrasound‐driven piezoelectric devices can convert self‐generated electricity to ultrasound waves back for various applications such as 3D ultrasound imaging or some communicative sensors while TENG devices lack this property. Hereby, using “receiver” can cover all the ultrasound‐driven piezoelectric devices as multifunctional convertors or generators for energy harvesting.

### 4.1 Ultrasound‐Trigged Piezoelectric Receivers

**Figure 5:**
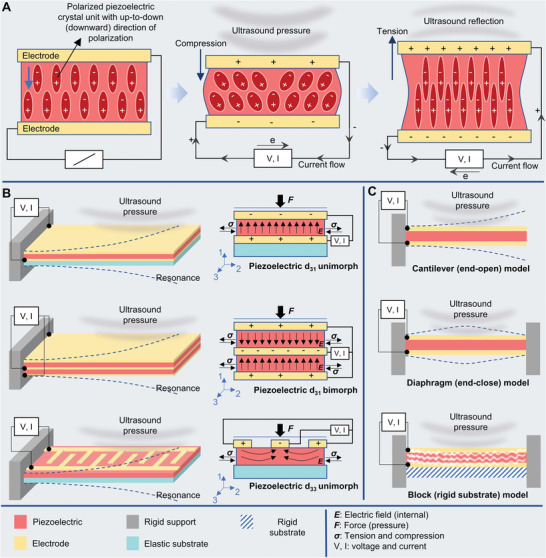
Schematic diagram of A) piezoelectric energy harvesting mechanism from deformation of polarized piezo crystal units by compression and tension through ultrasound pressure, B) PENG devices are based on configuration and based on the direction of electric field and stress/compression including d_33_ and d_31_ modes and bimorph and unimorph structures, C) different rigid support configurations of piezoelectric devices with different mechanical motions including the cantilever (end‐open) model with asymmetric resonance, diaphragm (end‐close) model with symmetric resonance, and block (rigid substrate) model with vibration.

The US‐PENGs are devices that convert mechanical vibrations of ultrasonic waves into electrical energy using the piezoelectric effect at the nanoscale.^[^ ^113^, ^114^ ^]^ The US‐PENGs often consist of 2D piezoelectric materials such as tungsten diselenide (WSe_2_) or molybdenum disulfide (MoS_2_), piezoelectric ceramics like barium titanate (BTO), zinc oxide (ZnO) nanowires or lead zirconate titanate (PZT) nanorods, and piezoelectric polymer such as polyvinylidene fluoride (PVDF), sandwiched or covered by electrodes and integrated onto a flexible/rigid substrate.^[^ ^115^, ^116^, ^117^ ^]^ **Figure** **5**A presents the electric generation mechanism of piezoelectric receivers when exposed to ultrasonic vibrations; the piezoelectric materials within experience mechanical stress induce the generation of an electric charge that can be utilized to power small electronic devices or sensing. The piezoelectric receivers can be classified based on configuration and based on the direction of the electric field and stress/compression. The d_33_ and d_31_ modes indicate parallel direction and lateral direction of the electric field with stress/compression respectively. In addition, the bimorph consists of two layers of piezoelectric material that are bonded together with opposite polarization and the unimorph consists of a single layer of piezoelectric material bonded to a passive layer (often a non‐piezoelectric material) on one side (Figure 5B).^[^ ^118^, ^119^ ^]^ Furthermore, the common support configurations of piezoelectric devices with different mechanical motions include an end‐open model with asymmetric resonance, an end‐close model with symmetric resonance, and a rigid substrate with vibration (Figure 5C).^[^ ^120^, ^121^ ^]^ This energy‐harvesting capability holds promise for self‐powered systems and wireless sensor networks, such as ultrasound scanners. By utilizing UMM structures, there is potential to enhance efficiency and power output, enabling the development of self‐powered medical implants or wearable devices.

**Figure 6:**
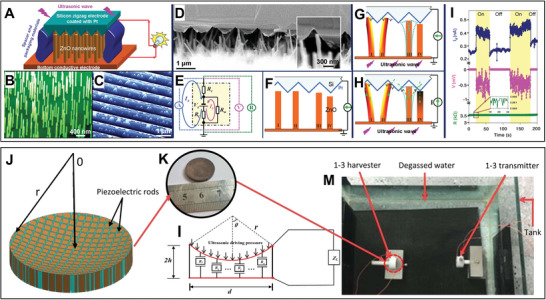
Novel architectured US‐PENGs for proposed energy harvesting. A) Schematic diagram showing the design and structure of the US‐TENG that aligned ZnO NWs grown on a solid/polymer substrate covered by a zigzag electrode, B) aligned ZnO NWs grown on a GaN substrate with a C) zigzag‐trenched electrode, D) a cross‐sectional SEM image of the nanogenerator which is composed of aligned NWs and the zigzag electrode, and E) equivalent circuit of the nanogenerator and the setup; F–H) schematic illustration of the zigzag electrode and the four types of NW configurations and the mechanism of the nanogenerator driven by an ultrasonic wave through NWs, and I) measured output (I, V, and R respectively) when the ultrasound was turned on and off purposely. Panels (A–I) adapted with permission from ref. [122], copyright 2007, Science. J) Schematic illustration and K) photo of 1–3 piezoelectric composite harvester of gradually varying thickness. L) The equivalent circuit model of the harvester and M) experimental setup. Panels (J–M) adapted with permission from ref. [123], copyright 2018, American Institute of Physics.

Some presented UMMs have been incorporated into receivers to augment the segmentation of ultrasound waves and their subsequent conversion into electrical energy. The configuration of nanostructures within nanodevices, like nanowires, nanotubes, or nanosheets, plays a pivotal role in advancing the efficacy of energy harvesting procedures. As shown in **Figure** **6**A, a DC nanogenerator based on ultrasound was developed, and fabricated with vertically aligned zinc oxide nanowire arrays that were sited beneath a zigzag metal electrode with a small gap (Figure 6B,C); the ultrasound wave (≈41 kHz and ≈3 MHz) drives the electrode up and down to bend and/or vibrate the nanowires (Figure 6D–I) and approach presents an adaptable, mobile, and cost‐effective technology.^[^ ^122^ ^]^ The ultrasound receivers are reported with a variety of structures; a wideband ultrasonic energy harvester using 1–3 piezoelectric composites with an inward‐curved circle structure and parallel‐connected oscillator arrays, was studied that can scavenge the ultrasonic energy in a range of frequencies while strongly depends on its thickness variation (Figure 6J–M).^[^ ^123^ ^]^ A wide of ultrasound receivers were designed with optimized structures such as resonant multilayer cantilevers,^[^ ^124^ ^]^ inward‐curved antennae,^[^ ^125^ ^]^ and membranes with coupled mechanical oscillators.^[^ ^126^ ^]^

Following the majority of developing piezoelectric generators based on ultrasound, piezoelectric microelectromechanical systems (MEMS) devices are fabricated by micromachining technologies for many applications. Most MEMS energy harvesters absorb ambient vibrations or sound waves by using thin vibrant/resonator films, which are fabricated through deposition techniques such as sol‐gel spin coating, screen printing, and sputtering. Many works have demonstrated the developments of MEMS.^[^ ^127^ ^]^ For example, a novel MEMS energy harvester has been designed for charging implanted biomedical devices, It is excited by externally supplied ultrasonic waves and features three mechanical degrees of freedom (3‐DoF MEMS) to allow energy to be harvested irrespective of the alignment between the harvester and an ultrasonic transmitter under 36 kHz.^[^ ^128^ ^]^ Furthermore, a 4‐degree‐of‐freedom (4‐DoF MEMS) resonant was presented that relatively generated more output at ≈25 kHz.^[^ ^129^ ^]^ New generations of MEMS were realized that showed great potential for powering devices such as implantable medical devices. Optimizing the ratio of the piezo‐layer thickness to the substrate/support layer thickness is effective, as well as selecting a practical design that enhances the sensitivity of the device based on ultrasound. Experimental investigations have been conducted with PZT and silicon layers at 88 kHz underwater, revealing a dependence of transferred power magnitude on the size of the receiver and operating frequency.^[^ ^130^ ^]^

**Figure 7:**
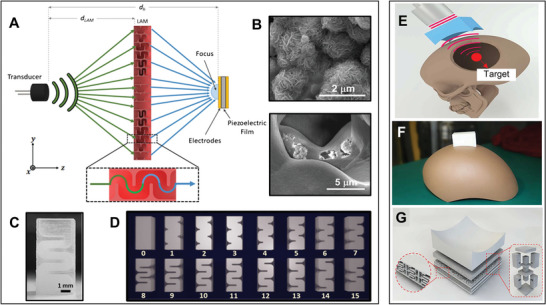
Transmissive and complementary UMMs for focusing and penetrating ultrasound waves toward US‐ETs. A) Schematic design of the concept of boosted acoustic energy harvesting consuming a LAM, B) scanning electron microscopy and microscopic images of MoS_2_ nanoflowers (top) and MoS_2_ nanoflowers embedded PVDF film, C) a photograph of a single meta‐brick, and D) 16 meta‐brick designs, each capable of providing individual amplitude and phase modulations. Panels (A–D) adapted with permission from ref. [60], Springer Nature Limited. E) Schematic diagram of phase‐modulated complementary UMM in ultrasound brain focusing on the intact skull, F) 3D printed PCMM for ultrasound propagation through a skull and G) geometry of the PCMM, bottom of the PCMM, and middle of the PCMM respectively. Panels (E–G) adapted with permission from ref. [24], copyright 2022, Wiley‐VCH.

Recently, UMMs have been explored based on coiling space lenses or resonance‐based designs such as using Helmholtz resonator structures for focusing ultrasound energy on the US‐PENGs. A more advanced level of UMMs was experimentally demonstrated by inspiration from the focusing concept, a novel system that projects the ultrasound (40 kHz) holographic output onto the harvester using a transmissive labyrinthine acoustic metamaterial (LAM) as shown in **Figure** **7**A, with internal geometries of meta‐brick that take the form of a space coiling pathway (Figure 7C,D); maximum power density value of 18.2 mW m^−2^ and a maximum power rate of 272% were gained using the LAM with a nanocomposite harvester film made by inculcating MoS_2_ nanoflowers in a PVDF matrix film (Figure 7B).^[^ ^60^ ^]^ Furthermore, UMMs can be used as a mask or aberration layer on the probe, allowing sound waves to pass through the layer without losing energy through impedance‐matched that matching material is called a couplant. A phase‐modulated complementary UMM (PCMM) based on the principle of impedance matching, which enables ultrasound (113 kHz) to penetrate the bone was designed that is made through 3D printing and corrects bone aberrations (Figure 7E).^[^ ^24^ ^]^ The top part of PCMM is formed for a snug fit to the skull (Figure 7F). The middle section contains a straight network with two Helmholtz resonators linked to the main volume by narrow slits, and the bottom part is phase‐modulated based on Helmholtz resonators and space coil portion screens (Figure 7G).

**Figure 8:**
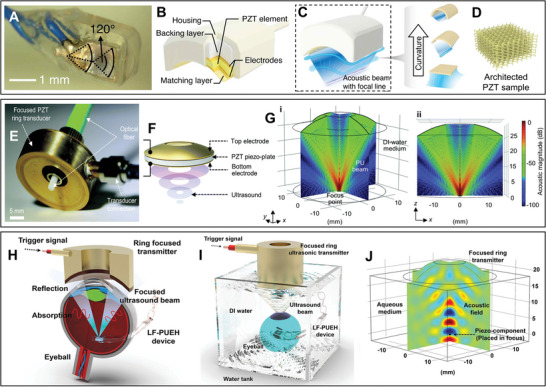
Architecture and function of fabricated focused ultrasound transducer probes based on UMMs. A) Optical and schematic images of a 3D printed miniaturized ultrasound transducer, B) a focused micro‐PZT element, C) schematic of the fabricated miniatured ultrasound transducer with curved PZT elements and comparing micro‐curved stave elements with different curvatures of ultrasound waves, and D) a penta‐mode metamaterial structure of piezoelectric elements. Panels (A–D) adapted with permission from ref. [131], copyright 2023, Springer Nature Limited. E) An Image of a focused ring piezo‐transducer configured with an optical fiber, F) a schematic diagram illustrating the major concept of a focused PU transducer, and G) a simulated PU field generated by a focused ring piezo‐transducer with an inner diameter of 10 mm, an outer diameter of 28 mm, a focal length of 25 mm, and a center frequency of 3.3 MHz. Panels (E–G) adapted with permission from ref. [132], copyright 2021, The Royal Society of Chemistry. H) Schematic setup and I) profile of the implanted mm‐scale flexible LF‐PUEH into an excised eyeball to mimic an implant situation, and J) the simulated ultrasound field of a focused transducer. Panels (H–J) adapted with permission from ref. [133], copyright 2019, Wiley‐VCH.

The performance of US‐ET is strongly depending on the geometries of active elements of the transducer as well as the architecture of receivers. In addition, fabrication strategies are considered because of the physical properties of piezoelectric materials such as fragility. Hereby, a novel 3D printed carved piezoceramic micro‐transducer was introduced as shown in **Figure** **8**A,B, to produce high power of focused ultrasound at MHz range (Figure 8C), that contains a penta‐mode metamaterial structure of piezoelectric elements (Figure 8D), for biomedical applications such as localizing ultrasound energy onto implanted microdevices.^[^ ^131^ ^]^

A more facile fabrication of a focused ultrasound transducer consumes the curved (bowl‐shaped) ring as the top and bottom electrodes to sandwich the piezo material. A recent research work demonstrated a small focused ultrasound transducer was used in biomedical applications as exhibited in Figure 8E. The PZT ring plate with silver electrodes on both sides was fixed into a curved brass housing (Figure 8F), and the brass housing was connected to a ground connection for simulating the ultrasound pressure field created through a focused ring piezo‐transducer (Figure 8G).^[^ ^132^ ^]^ Furthermore, another focusing transducer was applied to pass and localize the ultrasound wave on an implanted lead‐free piezoelectric ultrasonic energy harvester (LF‐PUEH) that shows the feasibility of ultrasound‐induced wireless energy harvesting for potential retinal electrical stimulation application (Figure 8H,I) and the sufficient focused ultrasound was confirmed by simulated ultrasound field of a focused transducer (Figure 8J).^[^ ^133^ ^]^

### 4.2 Ultrasound‐Trigged Triboelectric Nanogenerators

**Figure 9:**
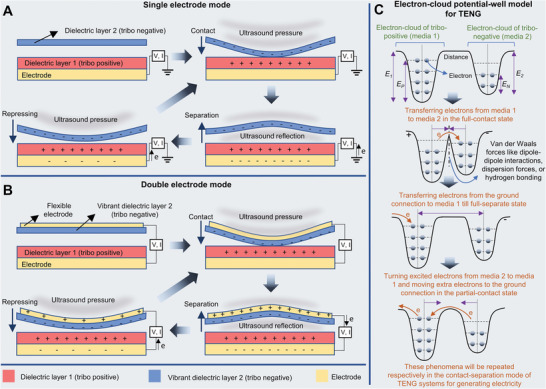
Schematic diagram of energy harvesting mechanism and common basic modes for the US‐TENG. A) Single electrode and B) double electrode modes of US‐TENG. C) An electron‐cloud potential‐well model for showing the mechanism of triboelectric generating in the contact‐separation mode; E_P_ and E_N_ present the occupied energy levels of electrons of tribo‐positive and tribo‐negative materials; E_1_ and E_2_ represent the potential energies for electrons to escape from the potential‐well.

Triboelectric receivers based on ultrasound perceptibly use mechanical vibrations as a driving force to induce triboelectric charging and generate electrical energy.^[^ ^134^ ^]^ This concept combines the principles of triboelectricity and ultrasound to enhance the performance and functionality of nanogenerators. Triboelectricity is the phenomenon of electric charge generation through the contact and separation of materials with different surface potentials.^[^ ^135^ ^]^ When two materials with different electron affinities come into contact and then separate, a transfer of electrons occurs, resulting in the generation of an electric charge. In the context of TENGs, ultrasound waves are utilized to induce mechanical vibrations in a system, and these vibrations can create relative motion between the contacting materials. This cyclic process leads to the generation of electrical charges due to the triboelectric effect, and the harvested energy can be used to power small electronic devices or stored in energy storage systems.^[^ ^136^, ^137^ ^]^ **Figure** **9**A,B illustrate the electric energy harvesting working mechanism of TENGs through contact‐separation of two media under ultrasound wave pressure in single‐electrode and double‐electrode modes respectively. The tribo‐layers close each other until a full‐contact state that is resulting electrons moving from tribo‐positive to tribo‐negative. Therefore, the electron vacancies are occupied by receiving electrons from the ground connection to tribo‐positive till a full‐separate state. Following, the excited electrons move from tribo‐negative to tribo‐positive (through media surfaces) and retake further electrons by ground in a semi‐contact condition. These steps are repeated respectively according to the frequency of mechanical contact‐separation movement. Additionally, Figure 9C presents an electron‐cloud potential‐well model for demonstrating the mechanism of triboelectrification generating between tribo‐positive media and tribo‐negative media with different work functions. In all types of US‐TENGs, the contact‐separation is between a vibrant media and a rigid media.^[^ ^138^ ^]^

The surface charge density of tribo materials is the most important parameter that determines the output performance of TENGs and can be enhanced via selecting contact material with larger differences in electron affinity, chemical treatment like functionalization, middle layer insertion such as ferroelectric and piezoelectric materials, circuity assistance, or surface modification in micro‐/nanostructures and structural optimization by applying metamaterial structures.^[^ ^139^, ^140^ ^]^ The use of ultrasound from ambient sources as a driving force in The TENGs offers several potential advantages in various fields. The exploration of design optimization of US‐TENG is still an area of active research to improve the output performance for practical applications.^[^ ^141^, ^142^ ^]^

**Figure 10:**
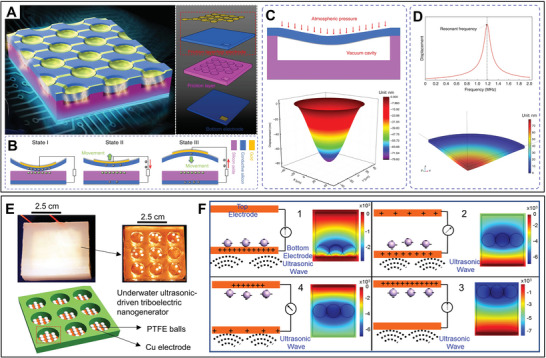
Structural architectured US‐TENGs for proposed ultrasound energy transfer technologies. A) Structural schematic illustration and B) mechanism of the µTUD under ultrasound vibration; C) a cross‐sectional graphic of the µTUD under atmospheric pressure for analytical modeling with a 3D mapping of static displacement surface of membrane‐based and D) displacement result of the µTUD in the frequency spectrum by numerical analysis with a static displacement surface for membrane. Panels (A–D) adapted with permission from ref. [45], copyright 2020, Springer Nature Limited. E) Photograph and schematic diagram of packaged and inter structure of underwater ultrasound‐based TENG, and F) working principle of the device in four steps through contact‐separation mode. Panels (E,F) adapted with permission from ref. [143], copyright 2017, Elsevier.

US‐TENGs still display a low ultrasound energy conversion efficiency, especially in deep underwater, owing to the pressure generated by water. Nonetheless, beyond the typical multilayer structure of US‐TENGs, architected materials can exhibit further functional structures for ultrasonic responsiveness for enhancing the elastic mechanical vibration of the tribo‐layer against ultrasound. Herein, a miniaturized ultrasound energy converter, that is, a micro triboelectric ultrasound device (µTUD) with high accuracy, has been developed for signal communication underwater. It is fabricated in a double electrode mode with a 50 µm‐sized diaphragm or resonant membrane structure (**Figure** **10**A,B), enabling working frequency at MHz with 63 kPa@1 MHz ultrasound input (Figure 10C,D).^[^ ^45^ ^]^

To extend the feature of metamaterials, a structural device with active architected materials is demanded to eliminate collapse in the regular contact‐separation movement of a vibrant layer of US‐TENG, through utilizing unattached micro particles in the empty microchannel. Therefore, another US‐TENG was developed as illustrated in Figure 10E, to be utilized in underwater applications such as animals and geological sound/movement by using spherical pellets as the media in cylindrical holes for performing the contact‐separation operation during ultrasonic wave excitation for energy harvesting at 80 kHz (Figure 10F).^[^ ^143^ ^]^

## 5 Applications of US‐ET Based on Metamaterials Structures

Nanogenerators based on ultrasound have various potential applications and this review includes a common classification of them in this section, such as implantable and wearable medical devices, robotics, wireless communication, sterilization, and treatment. As technology advances and efficiency improves, we can expect to see further exploration and expansion of their applications in various fields. Initially, in **Table** **1** and **Table** **2** an encyclopedic list of developed US‐ETs based on PENG and TENG respectively in high frequencies with different media types and trend structures that show linkage to metamaterial science and engineering concepts. To give an informative review, these performance tables have been organized to detail the size of each ultrasound receiver device, the configuration of the structure, harvested power, applied frequency and intensity of ultrasound, probe distance, media type, and application/advantages of devices. The best‐performing US‐ET systems from 1987 to 2023 for PENGs and from 2017 to 2023 for TENGs were selected to highlight the current state of ultrasound‐driven generator works regarding the realization of responsive metamaterial connections. For each item, a brief elaboration of notable studies is described to depict these novel systems through unconventional architectures.

**Table 1:** Reported structural developments of US‐ET systems based on piezoelectric receivers through tissue/fluid, metal, or air mediums.

Media type	*f* and *I* of ultrasound	Probe distance	Harvesting performance	Applications /advantages and efficiency	Trend design	Device size	Year	Ref
Air	1 MHz	20 cm	–	Surface characterization	Focused lens with microporous structures	10 mm	1987	[187]
Tissue	100 kHz, 1.5 W cm^−3^	–	34 V	Power supply to implanted medical devices, E: 36%	PZTs in device of magnetostriction with cuboid‐space structure	D: 40 mm T: 3.5 mm	2003	[188]
Water	≈3 MHz	–	10 µW cm^−2^	DC output for powering nanodevices	Vertically aligned nanowire arrays under a zigzag metal electrode	≈2 mm^2^	2007	[122]
Metal	1 MHz	57 mm	0.25 W	Pressure sensors and wireless communications	Block of solid steel barrier with resonant crystals	D: 2.54 cm	2007	[189]
Water	673 kHz, 94 mW cm^−2^	40 mm	3 V	Focusing ultrasound for implantable devices, E: 27%	Matching layer of graphite on PZT disk	D: 15 mm T: 1.3 mm	2009	[47]
Water	650 kHz, 94 mW cm^−2^	5 mm	6.75 V	Focusing ultrasound for implantable devices, E: 39.1%	Silver electrode with coiling space structure	0.53 cm^3^	2010	[160]
Air	36 kHz, 100 V	–	0.7 V, 12.6 nW (20 s)	Powering microdevices	3‐degree of freedom MEMS	≈4 mm^2^	2012	[130]
Air	55 kHz	230 mm	≈4 V	Focusing ultrasound for energy harvesting	Elastic arrayed 2D lattice structure	D: 7 mm T: 0.2 mm	2013	[190]
Air	1.07 MHz	–	–	Power and signal delivery to hearing aids, E: 45%	Square pillars of PMN‐PT surrounded by epoxy matrix	D: 5 mm T: 1.2 mm	2013	[191]
Tissue	255 kHz	23 mm	3.25 mW	Focusing ultrasound for implantable devices, E: 21%	Utilized a focused matching layer	15.7 cm^3^	2013	[192]
Air	17 kHz	100 mm	248 µW	Reducing impedance mismatch of UET systems air‐based, E: 0.68%	Stepped horn structure for transducer & receiver	–	2013	[193]
Water	≈200 kHz, 13 mW cm^−2^	5 mm	Vibration amplitudes: 9.9 µm	Biomedical power applications, E: 3%	MEMS with coupled mechanical oscillators	40 × 40 × 3 mm^3^	2014	[126]
–	25 kHz	50 mm	X: 24.7 nW Y: 19.8 nW Z: 14.5 nW	Powering implanted biomedical devices	MEMS with 3 degrees of freedom design	4 mm^2^	2014	[194]
Water	1 MHz	105 mm	25 mW	Deep implanted medical devices, E: 1.6–2.3%	Concave structure for ultrasound focus	2880 mm^2^ 50 mm^2^	2014	[195]
Water	47. 7 kHz	–	41 µW (cm^3 ^s^−1^) ^−2^	Biomedical implants and sensors	Focused piezoelectric source with concave structure	–	2014	[196]
Water/tissue	40.43 kHz	22 mm	49 µW	Implantable microdevices, E: 0.096%	MEMS membrane‐type structure	3.2 × 3.2 mm^2^	2014	[163]
–	25 kHz	–	X: 50.9 nW Y: 60.6 nW Z: 14.3 nW	Powering implanted biomedical devices	MEMS with 4‐degrees of freedom design	16 mm^2^	2015	[129]
Air	40 kHz	20 mm	7.24 mW	Low‐power applications, E: 100%	Conical Al resonator on the piezoelectric elements	D: 16 mm	2016	[50]
Water/tissue	1 MHz, 1 W	10–15 mm	600 mW	charging implanted medical devices, E: 20%	Assembling PZT circular disc in Al housing	D: 70 mm	2016	[197]
Water	PUEH 1: 350 kHz, PUEH 2: 285 kHz, 1 mW cm^‐2^	10 mm	1: 34.4 nW 2: 84.3 nW	Implantable biomedical devices, E: 40%	MEMS with diaphragms connected in parallel	1: 4.67 mm^2^ 2: 2.06 mm^2^	2016	[161]
Water/tissue	0.1–1 MHz, 1 mW cm^−2^	5 mm	3.75 µW cm^−2^	Self‐powered implants	MEMS with Dicing chips on diaphragm structure	25 mm^2^	2016	[162]
Water	5 MHz, 100 V	–	–	Compressive 3D ultrasound imaging using a single sensor	3D plastic ultrasound mask with a jagged checkered pattern	D: 12.7 mm (mask size)	2017	[174]
Tissue	1.314 MHz, 23 mW mm^−2^	105 mm	15 V	Nerve stimulation	Air‐backed structure to increase aperture efficiency	2 × 3 × 6.5 mm^3^	2018	[198]
Tissue	>20 kHz	–	3.6 V	Powering implantable devices	1‐3 piezoelectric composite with inward‐curved circle structure	D: 20 mm	2018	[123]
Metal	440 kHz	6.3 mm	3.9 V	Structural health monitoring of solid metal structural components, E: 33%	Assembled and miniaturized sensor embedded within metal	11.9 cm^3^	2018	[199]
Air	40–70 kHz	–	≈4.3 µW, ≈0.7 V	Ultrasound wave focusing and energy harvesting	3D‐printed lens (rectangular array of cylindrical nylon stubs with varying heights)	7 × 0.4 mm^2^	2019	[200]
Tissue	1): 562 kHz, 2): 650 kHz, 185 mW cm^−2^	10 cm	1): 108 µW, 2 V, 54 µA, 2): 171 µW, 3 V, 57 µA	Photodynamic therapy (cancer treatment), E of 1): 1.7% E of 2): 2.7%	Embedded PZT in a U‐shape backing structure and integrated µ light	1): 2 × 2 × 2 mm^3^ 2): 2 × 4 × 2 mm^3^	2019	[201]
Water	88 kHz, 322 mW cm^−2^	20 mm	0.7 mW	Powering implantable device, E: 0.33%	MEMS with diaphragm structure	2 × 2 mm^2^	2019	[127]
Water/tissue	304 kHz, 300 V	20 mm	45 mW cm^−2^	Retinal electrical stimulation	1‐3 piezoelectric composites, parallel‐connected and checkered‐arrayed	1 × 1 × 0.6 mm^3^	2019	[133]
Water/tissue	350 kHz, 300 V	23 mm	2.1 V, 4.2 µA	Flexible implantable wireless generator, E: 0.0063%	Dicing 1‐3 piezoelectric composite, parallel connected and checkered‐arrayed	7 × 7 (1.5 × 1.5 mm^2^)	2019	[157]
Air	40–50 kHz	–	4.55 V	For highly dense piezoelectric energy harvesting	2D octagonal phononic crystals, (5 × 7)	3.2 × 3.2 cm^2^	2019	[186]
Air	55.5 to 56.5 kHz	10 cm	Max: 16.2 V	Output performances of a PnC‐based piezoelectric energy harvesting system	Designed a phononic crystal (PnC) with a defect, (5 × 7 supercells)	Each cell: 27 × 27 × 12 mm^3^	2020	[77]
Air/water/ tissue	15–30 kHz	–	–	Non‐destructive testing, biomedical implants	3D printed focusing ultrasound lens with periodic space band‐structure	Lens size: 156 × 324 × 208 mm^3^	2020	[202]
Air	40 kHz	10–30 cm	35–10 mW	Focusing ultrasound on powering tangible and wearable devices, E: 0.0084%	Arrayed focal points of transmitter (4 by 4 points)	–	2021	[203]
Water/tissue	3.6 MHz	1.85 cm	9.43 mW	Focusing ultrasound in implantable neurostimulators, E: 2.66%	Focused probe and receiver by press technique	D: 6 mm	2021	[204]
Water/tissue	3.3 MHz	10 mm	≈11.08 µW	In implantable selective wireless energy transfer at MHz frequencies, E: 5%	Printed grating space 2D zigzag (by dicing‐and‐filling strategies)	25 × 10 × 12 cm^3^	2021	[170]
Water/tissue	1.1 MHz	20 mm	6 mW	Powering biomedical implants, E: 0.14%	Focused ultrasound with dicing parallel arrayed	≈1 mm^3^	2021	[205]
Water/air/polymer	55 kHz	–	–	Transmission ultrasound power from barrier without energy loss, E: 0.053%	Complimentary meta‐layer (CML) with resonator space coiling structure	–	2021	[51]
Water/tissue	1 MHz	5 cm	13.5 V	Implantable nerve stimulators	BZT‐BCT nanowire on PDA film	1.5 × 1.5 cm^2^	2021	[206]
Water/tissue	40 kHz, 0.6 W cm^−2^	18.5 cm	21 V, 2 mA, 304 mW cm^−2^	Implantable devices, E: 0.051%	Unidirectional 3D interconnected ceramic–polymer topology as PZT framework (wood)	3 × 3 cm^2^	2021	[166]
Water/tissue	3.3 MHz, 100 V	12 mm	≈21.3 mW cm^−2^	Wireless multifunctional implants	Dicing 1–3 piezo composite, parallel‐connected and checkered‐arrayed, (1 × 13 arrays)	1 × 13 (1.5 × 1.5 mm^2^), 2 mm of space	2021	[132]
Water/tissue	1 MHz 212 mW cm^−2^	30 mm	280 µW	Brain stimulation, analgesia applications, E: 46–70%	Connected dicing piezoelectric elements in 6 × 6 arrays	13.5 × 9.6 × 2.1 mm	2022	[207]
Water/tissue	100 kHz, 25 mW cm^−2^	5–10 mm	≈13.13 mW	Neuro‐prosthetics, Wireless power supply, and drug delivery; E: 0.62%	Resonant multilayer structure with parallel‐connected air hole array	1 × 1 cm^2^	2022	[164]
Water	3 MHz, 17.85 mW	25 mm	18.8 µw	Multifunctional implantable devices, E: 0.236%	Vibrant cantilever piezoelectric micromachined	2.55 cm^2^	2022	[124]
Water/tissue	2.08 MHz	10–20 mm	22.4 V, 0.145 W cm^−2^	Lead‐free piezo, diagnostics, therapy, and real‐time monitoring; E: ≈20%	Connected dicing 1–3 piezo‐composite elements in 4 × 5 arrays	4 × 5 (1 × 1 mm^2^), 2 mm of space	2022	[208]
Air	0.1–20 kHz, 50–120 dB	0–120 cm	≈60 mV (maximum)	Human–machine interaction, speech recognition; 98% accuracy	Vibrant multilayer structure (layer‐particles‐layer)	Active area: 30 × 30 × 0.35 mm^3^	2022	[209]
Water/tissue	100 kHz, 0.7 W cm^−2^	≈8 cm	0.3 mW cm^−2^, 14 V, 40 µA	Tissue engineering applications	Rolled multilayer structure composed with shell–core nanowires	1.5 × 1.5 cm^2^	2022	[209]
Water/tissue	1 MHz, 1.56 mW cm^−2^	2 cm	≈12 V	Spinal cord injury treatment by electrical stimulation	Rolled piezoelectric scaffold with 3D printed microchannels on the nanofiber film	L: 17 mm D: 2 mm	2022	[210]
Water/tissue	28 kHz	0.5–4.5 cm	≈10 mW	Implantable medical devices, reducing impedance	Vibrant membrane and multilayer structure	4 × 4 mm^2^	2022	[134]
Water/tissue	400 kHz, ≈180 dB	3.5–13.5 cm	9.5–1.5 dB	High data‐rate intrabody communication	Resonator multilayer diaphragm	3 × 3 mm^2^	2022	[211]
Water/tissue	40 kHz, 0.2–0.5 W cm^−2^	10–50 mm	4 V cm^−1^ (0.3 W cm^−2^ and 10 mm)	Brain cancer treatment	Vibrant membrane and multilayer structure	2 × 2 × 0.1 cm^3^	2022	[168]
Water/tissue	3.3 MHz, 22.6 W cm^−2^	25 mm	4.3 V	Flexible retinal stimulating for biomimetic visual prostheses, E: ≈11.3%	Connected dicing 1–3 piezo elements (checkered pattern)	D: 12 mm	2022	[158]
Air	35.1 kHz, 67 V	–	58 mW, 82.4 mW	Ultrasound vibration energy harvesting, E: 4%	Jagged piezoelectric ultrasound actuator	D: 60 mm	2023	[212]
Water/air/tissue‐air	20 kHz, 100 V	Air: 80 cm, water: 12 cm, Tissue: 35 cm	Air: ≈2.5 V, water: 1.5 V, tissue‐air: 3 V	Flexible multifunctional and wearable ultrasound underwater sensor	Resonator multilayer structure	3 × 2 × 0.1 cm^3^	2023	[159]
Water/tissue	20 kHz 1 W cm^−2^	5 mm	532 mV	Injecting a biodegradable microdevice to relieve infection, and accelerating wound healing	Vibrant multilayer tube structure	2 × 0.2 × 0.05 cm	2023	[213]
Air	469 kHz, 5 V	50 mm	≈18 mV	Ultrasonic auto‐positioning and multimodal sensor intelligence in soft robot	Vibrant multilayer membrane structure	–	2023	[177]

*f*: frequency, *I*: intensity, *E*: efficiency, *D*: diameter, and *T*: thickness of the device.

**Table 2:** Reported structural developments of US‐ET systems based on triboelectric receivers through tissue/fluid, metal, or air mediums.

Media type	*f* and *I* of ultrasound	Probe distance	Harvesting performance	Applications/advantages and efficiency	Trend design	Device size	Year	Ref
Water	80 kHz, 1.4 W cm^−2^	–	0.36 W cm^‐2^	Underwater ultrasonic energy transfer, E: 13.1%	PTFE pellets in cylindrical holes of cubic acrylic plate	9 × 9 × 1.27 cm^3^	2017	[143]
Water/tissue	20 kHz, 1 and 3 cm^−2^	5 mm	3.8 mW, 6.4 mW	Powering implacable devices	Multilayer structure with vibrant PFA film	3.6 × 3.6 mm^2^	2019	[138]
Oil/tissue	1 MHz 0.7 W cm^−2^	≈30 mm	297 nW	Implantable devices and signal communication, E: 33%	Diaphragm/membrane channel microstructures	1 × 1 mm^2^ (50 µm)	2020	[45]
Water/tissue	70 kHz, 0.3 W cm^−2^	–	30 mW cm^−2^	Vagus nerve stimulator (Anti‐inflammatory therapy) based on hydrogel‐TENG, E: 10%	Hydrogel with 3D network structures	1.5 × 1.5 × 0.21 cm^3^	2021	[214]
Water/air/solids	77 kHz	2.4 cm	1.8 mV	Structural health monitoring	Layer–powder–layer structure	D: 23 mm	2022	[173]
Water	49 kHz, 0.3 W cm^−2^	1 cm	–	Underwater wireless communication	Parallel‐connected hexagonal prism of 6 TENGs	1 × 1 cm^2^	2022	[172]
Water/tissue	40 kHz	6 cm	8 mW	Recharging implant and underwater wireless communication, E: 4%	Multilayer TENGs on a printed passive pyramidical cubic base	1 × 1 cm^2^ (9 cm^3^)	2022	[46]
Water/tissue	20 kHz, 2 W cm^−2^	3 mm	≈4 V	Electrical subcutaneous microorganism elimination	Vibrant multilayer structure	1 × 2 cm^2^	2022	[215]
Water/tissue	20 kHz, 0.5 W cm^−2^	5 mm	4.51 V	Powering transient electronics and accelerating degradation by ultrasound	Vibrant multilayer structure, fabricating polymer composite layer with optimized porous size	2 × 2 cm^2^	2022	[216]
Air	0.145–9 kHz	5 cm	–	Frequency‐selective acoustic and haptic smart skin interface, E: 95% accuracy	Hierarchical macrodome/micropore/nanoparticle structure	–	2022	[178]
Tissue	40 kHz, 0.2–0.4 mW cm^−2^	3–5 cm	4 V cm^−1^	Treatment of brain cancer using electromagnetic fields via triboelectric	A membrane‐type device with a Vibrant multilayer structure	2 × 2 cm^2^	2022	[168]
Water/tissue	100 kHz, 2 MHz, 100 V	40 mm	0.26 V (maximum)	Fully flexible implantable devices	Vibrant multilayer structure with interlocking conical nanostructures	10 × 10 mm^2^ T: 0.66 mm	2023	[167]
Water/tissue	20 kHz, 1 W cm^−2^	1 mm	3.21 mW, 15.5 V	Flexible, powering implantable device, reducing the sound impedance of encapsulant	Vibrant multilayer structure, and fabricating a colloidal encapsulant polymer	3 × 3 cm^2^ T: 0.31 mm	2023	[169]
Air	20 kHz, 0.74 dB	9 mm	3.7 µW at 360 k‐ohm	Energy and information transmitting smart tag based on lector‐static induction nanogenerator	1D Helmholtz resonator megastructure	D: 20 mm	2023	[217]

*f*: frequency, *I*: intensity, E: efficiency, D: diameter, and T: thickness of the device.

### 5.1 Medical Devices

Biomedical nanogenerators based on PENG and TENG can generate electricity through biomechanical energy (by muscular movements such as heartbeat, joints, respiration, and stomach) and ultrasound energy sources. While the body's motions are typically inaccessible for occasional use, ultrasound offers a noninvasive, sustainable, and has potential applications in the field of biomedicine, as it is commonly used for diagnostic imaging, therapeutic purposes, and timely interventions. The US‐ETs are categorized as active energy harvesters by external sources that this sustained energy can be used to power implantable or wearable medical devices continuously, such as pacemakers, implantable defibrillators, neurostimulators, drug delivery systems, and biosensors.^[^ ^144^, ^145^, ^146^, ^147^ ^]^ By doing so, US‐ETs eliminate invasive surgeries for frequent battery replacements.^[^ ^148^, ^149^, ^150^ ^]^ Due to biosafety, according to the conventional limit by the FDA, the spatial‐peak temporal‐average intensity of ultrasound for continuous‐wave application should be less than 720 mW cm^−2^ for medical and biological applications.^[^ ^151^, ^152^ ^]^

A well‐designed device structure should be explored to achieve a portable, precise, and functional expansion of US‐ETs. The structure of the device will be optimized based on applications. For example, some parts of the human body surface or organs such as the brain, eyes, and heart are curved; hereby, many efforts have been focused on developing flexible nanogenerators that mainly use polymer materials as biocompatible thin‐film substrates.^[^ ^153^, ^154^ ^]^

Furthermore, metamaterial structures have been attracted previously for improving US‐ET device's properties and behaviors. For example,1‐3 piezoelectric composites of piezo ceramic with low‐density polymers such as epoxy resin have been widely consumed because of their superior properties to bulk piezoelectric materials, which have flexibility, higher energy conversion efficiency due to more electromechanical coupling coefficient, and low acoustic impedance to match the medium such as water or human organization.^[^ ^155^, ^156^ ^]^ A few research works were dedicated to this topic by engineering responsive meta‐structures of nanogenerators to provide well‐control and therapeutically relevant effects with instantaneous effective output.

A flexible and implantable ultrasound‐based electric stimulator with 1–3 type lead‐free piezo‐composite was fabricated in mm‐scale through a dice‐and‐fill technique that is capable of being implanted into eyeballs for retinal electrical stimulation; the piezo‐composite contains rectangular piece KNNS which is arrayed in checkered arrangement with epoxy to be driven by focused ultrasound to produce adjustable and higher electrical outputs.^[^ ^133^ ^]^ A little further, a 1–3 lead‐free piezo‐composite of F‐KNN and epoxy was investigated that employs a delicate architecture configured in the hybridization of piezo‐ultrasound (PU) and photoacoustic energy transfer for 3D twining wireless multifunctional implants; which show multi‐mode transmission advantages with high power and better resolution.^[^ ^132^ ^]^ However, a flexible ultrasound‐driven 1–3 piezo‐composite nanogenerator with no lead‐free was arrayed for a bio‐implantable wireless generator, that showed higher output performance than previously reported US‐PENGs even under non‐focused ultrasound. The structure of this flexible piezoelectric ultrasonic energy harvester was arranged 7 × 7 pillars of a composite of PZT and conductive silver‐epoxy and uses multilayered flexible electrodes with elastomer encapsulation.^[^ ^157^ ^]^ A more structural developed of 1–3 piezo‐composite, was presented as a retinal stimulator for evoking visual percepts in blind individuals. The device integrates a 2D piezo‐array with 32‐pixel stimulating electrodes in a flexible printed circuit that each piezo element can be ultrasonically and individually activated, thus, spatially reconfigurable electronic patterns can be dynamically applied via programmable focused ultrasound beamline.^[^ ^158^ ^]^

The wearable and flexible sensors are exceedingly in demand for health monitoring, recording physical activities, and indoor positioning applications. However, numerous structurally developed wireless wearable sensors are proposed. For example, an ultrasound‐driven flexible piezoelectric is developed with multifunction of communication, sensing, and positioning under high‐frequency and low‐frequency ultrasound. This membrane‐type device is agitated as a vibrating whole diaphragm, thus transmitting and receiving ultrasound energy.^[^ ^159^ ^]^

The metamaterials‐based micromachining technologies create opportunities in personal non‐invasive wearable and implantable healthcare devices. For example, a T‐shape MEMS coupled micromechanical oscillator with high deformations under ultrasound energy was designed that specifically can be one of the rectilinear stepwise actuators (forward and backward motions) for a medical implant application adjustment such as shunt regulating the rate of flow via a valve in treating hydrocephalus.^[^ ^126^ ^]^ In addition to all these structural advances of US‐TE devices, ultrasound transmission must be effectively captured toward the receivers, which requires metamaterials with architectural design. For example, through Gaussian excitation of the transmitter, a silver mask as an ultrasound thin‐coupling later with a coiling space structure was designed for focusing the ultrasound waves in medical applications with significantly high intensity and low ultrasound energy loss.^[^ ^160^ ^]^

**Figure 11:**
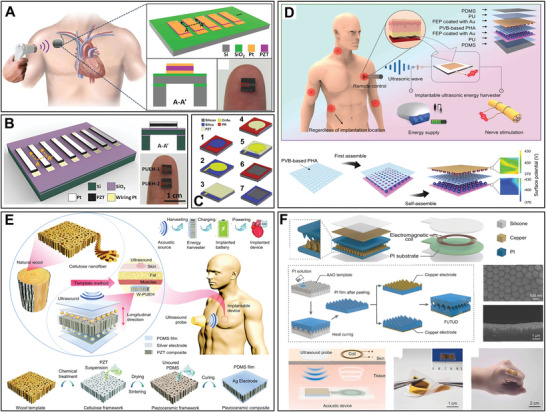
Energy harvesting for bio‐multiapplication through structural investigated US‐ET devices. A,B) a Schematic illustration of the MEMS‐based PUEHs with a cross‐sectional view of the multilayer diaphragm structure and the photograph, and C) a fabrication process of MEMS‐based ultrasonic receiver for wireless power. D) Overview illustration of H‐UEH system as implantable bioelectronics for energy supply and neuroprosthetics with the structure and process of preparing. E) Design and working principle of an implantable flexible W‐PUEH based on natural wood with lattice‐like cellulose channels, and manufacturing procedure using a modified template‐assisted sol–gel method. F) The structural design of the flexible, ultra‐wideband triboelectric ultrasonic device (FUTUD) boosted a flexible electromagnetic coil, with the fabrication process of the FUTUD and the SEM images of the nanostructure at the front and cross‐sectional view; in addition, the working mechanism of the implanted ultrasonic device with a view of the twisted device and patched on the skin are presented. Panel A adapted with permission from ref. [161], copyright 2016, Springer Nature Limited; panel B adapted with permission from ref. [162], copyright 2016, American Institute of Physics; panel C adapted with permission from ref. [163], copyright 2014, Elsevier; panel D adapted with permission from ref. [164], copyright 2022, Wiley‐VCH; panel E adapted with permission from ref. [166], copyright 2021, The Royal Society of Chemistry; and panels F adapted with permission from ref. [167], copyright 2023, Elsevier.

Significant variations in ultrasound frequency can notably diminish transferred power, especially when employing an energy harvester with a narrow operation bandwidth. This reduction occurs due to the substantial impedance mismatch between the transducers and the medium. To overcome these challenges, in recent years, there have been many meta‐structural design proposals for implantable and compact MEMS based on piezoelectric ultrasonic energy harvesters (PUEH). These proposals include a design featuring a resonant diaphragm array, which holds the promise of effectively recouping power and addressing efficiency loss. This is achieved by adapting to fluctuations in ultrasound frequency, an inevitability resulting from varying distances. This technology serves the purpose of charging the battery of pacemakers or other implantable biomedical devices, especially when operating at high frequencies. **Figure** **11**A illustrates a PUEH to enable self‐powered implantable biomedical devices, with the structural diaphragm design and materials. The results confirmed that by adjusting the resonant frequency output power can be increased due to resonation of the diaphragm structure under a tuned frequency. For instance, when the resonant frequency was shifted from 250 to 240 kHz, the output power surged 0.59 to 3.75 µW cm^−2^.^[^ ^161^ ^]^ Furthermore, another proposed PUEH comprises a micro‐scale diaphragm array with distinct geometric parameter designs, visually depicted in Figure 11B. The diaphragms in PUEH‐1 feature a larger length‐to‐width ratio intended to achieve a broadband property; however, this design compromises the energy harvesting performance. Conversely, the diaphragms in PUEH‐2 have a smaller length‐to‐width ratio and thinner thickness, aiming to attain both broadband property and robust energy harvesting performance.^[^ ^162^ ^]^ Thereafter, an investigated piezoelectric membrane type of MEMS was supplied with a high‐performance transmitter underwater or inside tissue at ≈40 KHz;^[^ ^163^ ^]^ the fabrication process and structure are roughly illustrated in Figure 11C. Recently, many efforts sufficiently demonstrated improving safe energy harvesting from medical devices inside the human body. For example, a multilayer ultrasonic‐induced piezoelectric harvester was developed based on the principles of stress concentration and electrostatic effects. This ultrathin, electrostatically assembled hybrid piezoelectric device leverages vertical strain enhancement of piezoelectricity by integrating a parallel‐connected air hole array between two electret films charged with opposing polarities. The resultant structure exhibits excellent piezoelectric properties and flexibility, thereby functioning as a high‐efficiency ultrasonic energy harvester (H‐UEH). This innovative configuration results in exceptional output and sound impedance matching, exceeding the power threshold requirements for bioelectronic devices and the current threshold necessary for nerve stimulation. This technology holds promise for the advancement of diagnostic and therapeutic applications in the next generation (Figure 11D).^[^ ^164^ ^]^

Although, metamaterials are artificially engineered and fabricated which exhibit superior properties not observed in nature, however, in the natural domain, certain 3D structures exhibit inherent arrangements, like wood with cylindrical local resonant periodic porous configurations. These naturally occurring structures can absorb ultrasound or vibration energy effectively.^[^ ^165^ ^]^ Hereby, as shown in Figure 11E, inspired by natural wood structures, an implantable wireless power transfer based on a flexible wood‐templated piezoelectric ultrasonic energy harvester (W‐PUEH) was presented with a unidirectional 3D interconnected framework of ceramic–polymer topology; that exhibits excellent power output under soft tissues at 40 kHz.^[^ ^166^ ^]^

In addition, new generations of wireless power transfers based on US‐TENGs are functionality developed and optimized by manipulating the structure through metamaterials. For example, an implantable, flexible, and ultra‐wideband US‐TENG was developed with ultrathin polymer film substrate and interlocking semi‐conical nanostructures to sense wide ranges of low and high frequency (20–200 kHz, 0.2–10 MHz) inside soft tissue; that was integrated with a flexible electromagnetic coil to transmit electrical signals wirelessly for bidirectional communication of real‐time monitoring in vivo physiological parameters (Figure 11F).^[^ ^167^ ^]^

The progress in US‐ETs has extended into diverse biomedical applications. Tackling brain tumors has proven to be a challenging endeavor. To address this challenge, an innovative implantable ultrasound‐powered tumor treatment device based on triboelectric technology was proposed. This membrane‐type device has a vibrant multilayer structure that operates by electromagnetically disrupting the rapid proliferation of cancer cells without causing any detrimental effects on normal neurons.^[^ ^168^ ^]^

In the structural design of devices, careful consideration of material selection is imperative. One critical aspect of biomedical US‐ET technology involves efficiently transmitting ultrasonic energy to implanted power generation devices while minimizing energy loss. A key technique to address this challenge is to reduce impedance mismatch in ultrasound transmission, by using polymers due to their sound impedance, which closely resembles that of surrounding human tissue. This similarity makes them well‐suited as encapsulation materials such as 2‐hydroxyethyl methacrylate (HEMA) to improve ultrasound transmission.^[^ ^169^ ^]^

**Figure 12:**
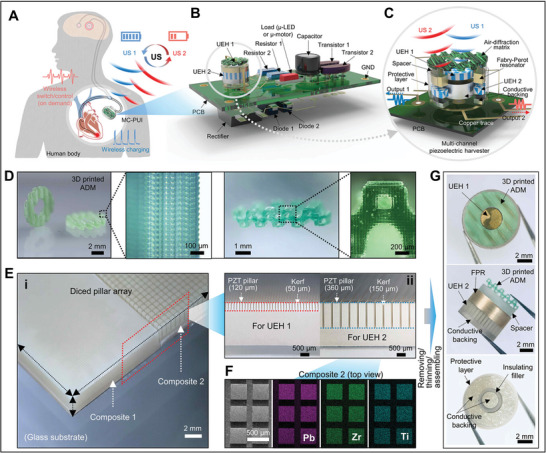
A selective ultrasound energy transfer for a potential multifunctional implantable device using a fabricated UMM. A–C) Schematics and principle structure of the piezo‐ultrasound implant (MC‐PUI) device with a hybrid water‐borne ultrasonic megastructure, D) optical images showing the 3D‐printed air‐diffraction matrix, E) The PZT pillar arrays after dicing and F) energy dispersive spectroscopy elemental mapping of the fabricated 1–3 composite, G) optical pictures display the top, side, and bottom views of the fabricated multichannel piezo‐harvester with HWAM. Panels (A–G) adapted with permission from ref. [170], copyright 2021, Wiley‐VCH.

With the increasing prominence of metamaterials in the US‐ETs for biomedical applications, a notable advancement includes a fabricated multichannel PU implant (MC‐PUI). This implant was intricately combined with a hybrid waterborne ultrasonic metastructure to enable selective wireless control via ultrasound frequency modulation in the megahertz range, depicted in **Figure** **12**A–C. Additionally, a meticulously optimized 3D‐printed air‐diffraction matrix and a half‐lambda Fabry–Perot resonator (Figure 12D–G), have been devised to capitalize on ultrasound selectivity at megahertz frequencies (1 to 3.3 MHz). The microchannels of HWAM are the key to realizing selective energy transfer due to forming an air scattering pattern. 1 MHz ultrasound effectively penetrates the HWAM, while at 3.3 MHz shows low transmission. This innovation offers heightened control capabilities for personalized medicine applications, specifically in vivo diagnosis and therapeutic interventions.^[^ ^170^ ^]^

### 5.2 Wireless Communication and Robotic

Ultrasound waves operating at high frequencies (>20 kHz) with short wavelengths exhibit extended propagation distances and strong directivity, characteristics extensively explored in various fields for detection and sensing applications, notably in underwater wireless sensing and robotic communication. The utilization of US‐TENG and US‐PENG as self‐powered sensors and actuators has garnered significant attention due to their potential. The exploring metamaterial concepts are delved to enhance their structure and augment output performance and efficiency at high frequencies. These innovative devices, utilizing metamaterial‐inspired designs, can harvest energy from ultrasonic waves originating from environmental sources such as vehicular traffic, machinery, or natural vibrations. They prove advantageous in scenarios where conventional power sources are limited or inaccessible. The harnessed energy can then be employed for diverse applications, including environmental monitoring, structural health assessment of buildings/bridges or infrastructure, wearable devices, robotics, and IoT applications.^[^ ^171^ ^]^

**Figure 13:**
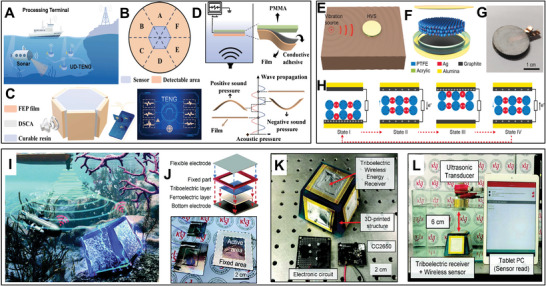
Designed US‐TENGs for effective underwater wireless communication and energy harvesting. A) The schematic of the self‐powered sensor network system for underwater ultrasonic wave detection and sound source localization. B) Detectable areas of the ultrasonic sensor and C) design concept of the ultrasonic sensor. D) Experimental setup and ultrasound propagation and vibration of the film. E,F) Schematic image of a self‐powered high‐frequency vibration sensor (HVS) for vibration sensing. G) Photograph of a typical HVS and H) a schematic showing the entire cycle of the electricity generation process of the HVS. I) Concept of underwater ultrasound energy transfer system employing the ferroelectrically boosted triboelectric receiver. J) Schematic exploded view, and K) image of the US‐TENG on a 3D printed quadrangular pyramid structure, with L) underwater Bluetooth wireless system in the water. Panels (A–D) adapted with permission from ref. [172], copyright 2022, Elsevier; panels (E–H) adapted with permission from ref. [173], copyright 2021, Elsevier; and panels (I–L) adapted with permission from ref. [46], copyright 2022, The Royal Society of Chemistry.

In recent years, numerous advancements have emerged in ultrasound‐based TENGs and PENGs, specifically designed for wireless communication and energy transfer systems. For instance, an easily fabricated and compact‐structured US‐TENG was introduced, employing a hexagonal prism made of UV curable resin created through 3D printing (**Figure** **13**B,C), and utilizing a separation‐contact mode (Figure 13D). This innovation aimed to actualize energy harvesting by increasing active area under high‐frequency ultrasound, thereby extending the potential applications of TENGs for in underwater military sensors (Figure 13A).^[^ ^172^ ^]^ A self‐powered broadband vibration sensor was engineered, employing a layer‐powder‐layer configuration based on a TENG, elucidated in Figure 13E–G with details. This sensor demonstrates the capacity to respond to high‐frequency vibrations ranging from 3 to 133 kHz, attributed to the internal composition of polytetrafluoroethylene (PTFE) and silver (Ag) microparticles integrated within a setup comprising double round graphite‐coated alumina ceramic sheets separated by an acrylic gasket. The alumina sheet possesses low sound impedance, optimizing to penetrate ultrasound. The graphite layers serve a dual purpose as the tribo‐positive material and electrode, while the PTFE particles function as tribo‐negative components, and the Ag particles act as electropositive triboelectric materials, collectively enhancing the triboelectrification effect (Figure 13H). Consequently, this sensor exhibits suitability for application in geological exploration across diverse materials.^[^ ^173^ ^]^ A more efficient model of US‐ET based on multilayer TENG with a ferroelectric boosting layer to enhance the triboelectric output and a flexible electrode. An enhanced model of the US‐ET has been proposed, utilizing a multilayer TENG integrated with a ferroelectric boosting layer to amplify the triboelectric output, as depicted in Figure 13J. This advancement involves the strategic placement of a ferroelectric single crystal possessing high remnant polarization beneath the triboelectric layer. This configuration maximizes contact electrification by finely tuning the work function of the triboelectric layer. In addition, to optimize the capture of ultrasound waves, five triboelectric receivers were positioned on a passive 3D‐printed trapezoidal pyramid structure (Figure 13K). This setup accommodates vibrant multilayer TENGs and incorporates multiple Bluetooth sensors dedicated to monitoring water quality (Figure 13I,L).^[^ ^46^ ^]^

**Figure 14:**
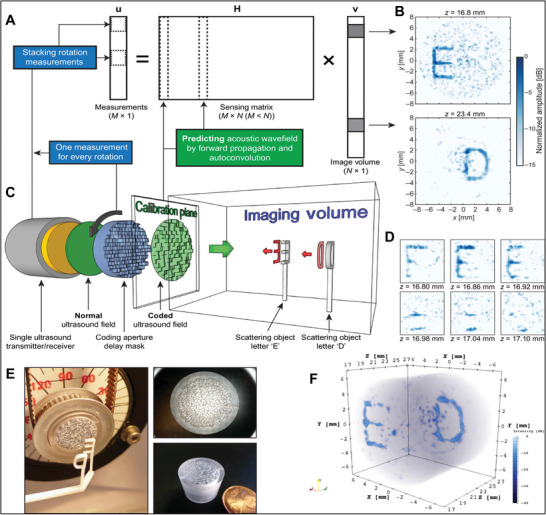
A compressive 3D ultrasound imaging by a single sensor. A) Plan sketch of the signal type involved in this model of compressive imaging. B) The outcome of solving part of the image vector v consuming an iterative least square method and C) an overview graphic of the thorough imaging setup. D) Reconstruction of the letter “E” in six adjacent z slices. E) Image showing the two 3D‐printed letters and the plastic coding mask with a rubber band for spinning the mask over the sensor. F) 3D rendering of the complete recreated image vector v, gained by BPDN. Panels (A–F) adapted with permission from ref. [174], copyright 2017, Science.

The 3D ultrasound imaging technology is a compelling advancement, particularly in the medical field, demanding an array of complex sensors to effectively capture reflected ultrasound waves by metamaterials. In response to this, a simplistically designed 3D plastic mask featuring a jagged checkered surface structure has been developed. This mask enables the generation of 3D imaging from compressed spatial ultrasound field measurements, utilizing only a single ultrasound sensor. The aperture mask is instrumental in ensuring that each pixel in the image is distinctly identifiable within a water medium. Through the implementation of this mask, the interference pattern within the stationary medium becomes rotatable by manipulating the aberration mask, facilitating multiple observations of the same object. Notably, this device offers advantages in terms of affordability, speed, simplicity, and compactness compared to prior sensor generations. For a comprehensive understanding, specific details are organized in **Figure** **14**A–F.^[^ ^174^ ^]^

**Figure 15:**
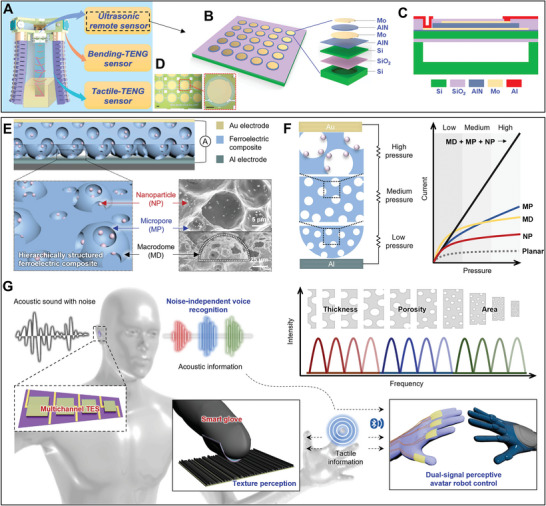
Robotic perception systems with ultrasonic sensors exhibiting human‐mimetic for advanced applications. A) A multifunctional robotic system with multimodal ultrasonic sensors, B) a detailed schematic of the piezoelectric micromachined ultrasonic transducers (pMUTs) array on a single chip to emit and receive the reflected ultrasonic waves, C) a cross‐sectional view of the multiple layers of a pMUT unit, and D) optical microscope image of the fabricated pMUT array (top view). Panels (A–D) adapted with permission from ref. [177], copyright 2023, American Chemical Society. E) Schematic of the triboelectric sensor (TES) using the hierarchical architecture of macrodome (MD), micropore (MP), and nanoparticles (NPs). F) Schematic of the dependency of each structural component of the hierarchical ferroelectric composite on pressure sensitivities of TESs. G) applications of TESs in numerous dynamic interfacing devices including noise‐independent voice recognition, texture perception, and dynamic motion recognition and interfacing using robotic hands; with a graph showing the frequency selectivity of TESs depending on the structural designs of hierarchical ferroelectric composites. Panels (E–G) adapted with permission from ref. [178], copyright 2022, Science.

TENGs and PENGs can be integrated into acoustic or ultrasound wave sensors for various applications in robotics. These sensors can detect and measure physical, chemical, or biological parameters by utilizing the energy harvested from ultrasonic waves such as gas sensors, liquid sensors, pressure sensors, and biosensors.^[^ ^145^, ^175^, ^176^ ^]^ A wide range of rigid and flexible smart metamaterial structures can be designed nobility for fabricating ultrasound‐based devices and satisfying specific factors such as output performance, compatibility, sensitivity, selectivity, stretchability, durability, washability, and self‐healing properties based on the device application like robotic functions.^[^ ^175^ ^]^ For example, a soft and sensitive robotic perception system with remote object positioning and a multimodal cognition capability was developed by integrating with a bending‐TENG sensor, a tactile‐TENG, and an ultrasound‐driven remote sensor (**Figure** **15**A). The Ultrasonic remote sensor has a membrane‐type structure that can distinguish the object's shape and distance by reflected ultrasound (Figure 15B–D). Thereby the robotic manipulator can be positioned to an appropriate position to accomplish object grasping, while the ultrasonic triboelectric sensors can capture multimodal sensory information at ≈470 kHz such as object top profile, shape, size, hardness, material, etc.^[^ ^177^ ^]^

Triboelectric technology compared to piezoelectric devices, is advantageous to create relatively larger structures in triboelectric systems, as this allows for more effective energy transmission, aligning well with applications such as underwater energy transmission.

In a recent study, a device featuring a hierarchical structure composed of macro‐domes, micropores, and nanoparticles within a ferroelectric composite, as illustrated in detail in Figure 15E, was developed with the primary goal of introducing a highly sensitive and frequency‐selective acoustic and haptic triboelectric sensor, as depicted in Figure 15F,G. This sensor exhibited a broad resonance frequency range spanning from 0.145 to 9 kHz, tailored for human–machine interfaces, highlighted in Figure 15G. The device demonstrated a remarkable accuracy of over 95% in voice recognition, surpassing conventional sensors, and showed applicability in various domains such as humanoid robots, wearable devices, and biometric systems.^[^ ^178^ ^]^

These research endeavors represent compelling and advanced structures through metamaterials that can be leveraged for high‐frequency operations across various functions of US‐ETs.

### 5.3 Nano and Micro Scale Therapy

Ultrasound‐activated piezoelectric nanomaterials (UA‐PENMs) as a converter ultrasound vibration to electric charge that can be utilized as an innovative electrical stimulator inside the body in a localized, wireless, and slightly invasive fashion^[^ ^179^ ^]^ for tumor/cancer therapy,^[^ ^180^ ^]^ neurostimulation and tissue regeneration,^[^ ^181^ ^]^ drug delivery systems,^[^ ^182^ ^]^ microorganism elimination, and wound healing.^[^ ^183^ ^]^ UA‐PENMs with enhanced piezoelectric coefficients can boost the efficiency of the therapy, which can be achieved by optimizing the composition and microstructures of their anisotropic shape. For example, a lattice heterostructure of Au@BTO was morphologically developed for sonodynamic therapy to facilitate the separation and migration of electric charge at the piezoelectric/metal interface, which produces effectively reactive oxygen species via redox reaction for killing the bacteria and cancer cells due to changing membrane potential; in addition, contributing to dermal wound healing (tissue regeneration) due to promoting cellular activity and enhancing blood flow, then resulting in migration of fibroblasts and macrophages for tissue repair.^[^ ^183^ ^]^ Other contrasting results were recorded on different nanoscale treatments and sterilization via ultrasound‐activated piezoelectric nanomaterials with developed structures such as core–shell, lattice, tube, rod/wire, and sheet.^[^ ^184^ ^]^

**Figure 16:**
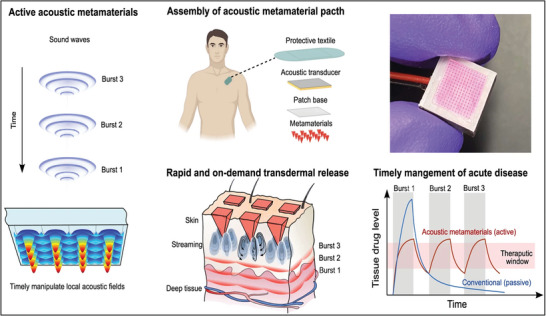
A schematic of the programmable and rapid transdermal drug delivery for opportunely controlling pharmacokinetics and handling the acute disease by an active acoustic metamaterial patch and a presentative image of an acoustic metamaterial patch. This panel was adapted with permission from ref. [185], copyright 2023, Springer Nature Limited.

Regarding fabricated novel structures of UA‐PENMs, the metamaterials at the nanoscale can be assisted in increasing the therapeutic performance. Also, at the microscale, a programmable and transdermal drug delivery with sharp pyramidal micro needles based on active metamaterial through ultrasound‐induced injection method at micro‐scale was realized for rapid and on‐demand acute disease management to effectively reduce the inability to timely administrate therapeutics and exactly regulate pharmacokinetics within a short time window. Indeed, the drug will be enhanced from the microneedles because of concentrating ultrasound stress in the tip of needles (**Figure** **16**).^[^ ^185^ ^]^ This item may help to reinvent ideas for the new generation of US‐ET in biomedical applications. Furthermore, ultrasound‐driven triboelectric technology offers a larger treatment scale through a flexible and bio‐adhesive multilayer device with the zigzag design of electrodes for instant wound sealing by electrical stimulation; that potentially can be a multifunctional device such as a nerve stimulator.^[^ ^186^ ^]^

## 6 Conclusions and Perspectives

In summary, the investigation of US‐TEs through metamaterials structures has demonstrated significant potential that opens up new avenues for sustainable energy solutions and presents innovative occasions across various applications. These metamaterials possess unique characteristic traits that allow the manipulation and regulation of ultrasound waves, qualifying effective propagation and reception of ultrasound waves over short distances without demanding physical connections or wiring, to facilitate efficient wireless power transmission of TENGs and PENGs.

The novel designs of metamaterials contribute to improving energy absorption, mitigation of energy loss due to wave reflections, and optimization of the mechanical‐to‐electrical energy conversion mechanism for wide applications such as wireless powering small‐scale devices. Ongoing research is actively directed toward refining the design and engineering of flexible and scalable metamaterial structures for integration into diverse devices to augment energy transfer efficiency, widen the operating frequency range, and enhance the overall performance of ultrasound‐based energy transfer systems. Material science advancements, fabrication methodologies, and modeling tools further propel progress in this domain. A primary challenge in the field lies in achieving high energy transfer efficiency over longer distances while ensuring low ultrasound power to maintain human body safety.

In this review, we present possible challenges with offered solutions via relevant references. Additionally, several vital research guidelines about improving the performance of the US‐ET based on UMMs are itemized and briefly explained within the below paragraphs.

The realization of the fundamentals and behavior of ultrasound waves, the physical, mechanical, electrical, and chemical characterization of US‐ET's composite is essential for achieving a beneficial result in manipulating the ultrasound waves with metamaterials. Already, many research works have been dedicated to characterizing and showing the properties of the sound and ultrasound waves in the variation ambient with different frequencies, intensities, and sources. However, choosing and optimizing the materials, and tuning the structure design are more accessible for enhancing the ultrasound‐capturing, resonation, frequency‐selectivity, and mechanical‐to‐electrical conversion through the characterization techniques.

Considering the material type in the fabrication of metamaterials is crucial for the transmission and absorption of ultrasound. Especially, the sound impedance and density are the main keys to achieving the purpose of the augmenting output. In some cases, the composition of metamaterials themselves has triboelectric or piezoelectric properties which is the generator of electricity in the US‐ET device. Hereby, its optimized properties such as stability, resonance capability, elasticity, sensitivity, and selectivity play a key role in US‐ET technologies. Furthermore, the selected materials must be concordant with the manufacturing methods of UMMs, and be compatible with mediums.

Typically, the SAMMs have a pivotal role in US‐ET applications. The SAMMs are used in the US‐ET devices for highly absorbing the ultrasound wave from the ambient to enhance the mechanical‐to‐electrical conversion ability of piezoelectric and triboelectric receivers under matched frequencies; the structure properties such as dimension and arrangement of unit cell/net are usually designed with the boosted vibrating and elasticity, for example, membrane or diaphragm type of devices and 1–3 piezo‐composite receivers.

The SFMMs are generally utilized for focusing the and augmenting mechanical pressure of ultrasound waves onto the target or receivers; the most cases, a concave objective with matched impedance is integrated in/on the ultrasound probe/horn for specific applications of energy harvesting.

Although the feasibility supplement of the US‐ET technologies is affected by the composite and structure of the UMMs, set‐up implementation of US‐ET systems also is the important key to increasing the performance, such as alignment position of the ultrasound source toward the device, as close as possible location of the US‐ET device in the medium to the ultrasound source, low thickness and matched impedance of the US‐ET's shield, and tuning of the frequency and intensity of ultrasound for purpose of resonation of generator. These optimizations encompass rigorous simulations and experimental validation processes to tailor the UMMs to the specific requirements.

## Conflict of Interest

The authors declare no conflict of interest.
